# Advancing Antidepressive Agents: Drug Discovery and Polymer-Based Drug Delivery Systems for Improved Treatment Outcome

**DOI:** 10.3390/biomedicines13051081

**Published:** 2025-04-29

**Authors:** Yufei Zhang, Zengyi Song, Hongxi Zhang, Haijiao Lin, Pu Xu, Zijia Li, Qingyun He, Binbin Wei

**Affiliations:** 1 Department of Pharmacy, Jinqiu Hospital of Liaoning Province, Shenyang 110122, China; 270596955@163.com; 2 School of Pharmacy, China Medical University, Shenyang 110122, China; szy1377@163.com (Z.S.); hoxii1121@gmail.com (H.Z.); lin13848956946@163.com (H.L.); 18635962182@163.com (P.X.); zijialee1360@163.com (Z.L.); hqy2839@163.com (Q.H.)

**Keywords:** depression, cause, mechanism, antidepressant therapy, polymer

## Abstract

Depressive disorder (a subclass of mental disorders) is characterized by persistent affective symptoms. Without timely therapeutic intervention, it leads to clinical deterioration manifested as reduced quality of life and may increase suicide risk in severe cases. Given its complex etiology, intertwined with intrinsic factors such as genetics and environment, and impacted by various issues such as first-pass effect and blood-brain barrier, the therapeutic efficacy of many antidepressant medications is limited for patients. Therefore, by delving into the exploration of novel antidepressant drugs and biomaterials, this review aims to offer fresh perspectives that may facilitate the discovery of innovative antidepressant medications and enhance their therapeutic outcomes. Notably, the review highlights polymers’ crucial role in enhancing antidepressants’ pharmacological efficacy and pharmacokinetic properties by optimizing their parameters, and they will undoubtedly become powerful tools in improving antidepressive outcomes in future research.

## 1. Introduction

**Figure 1:**
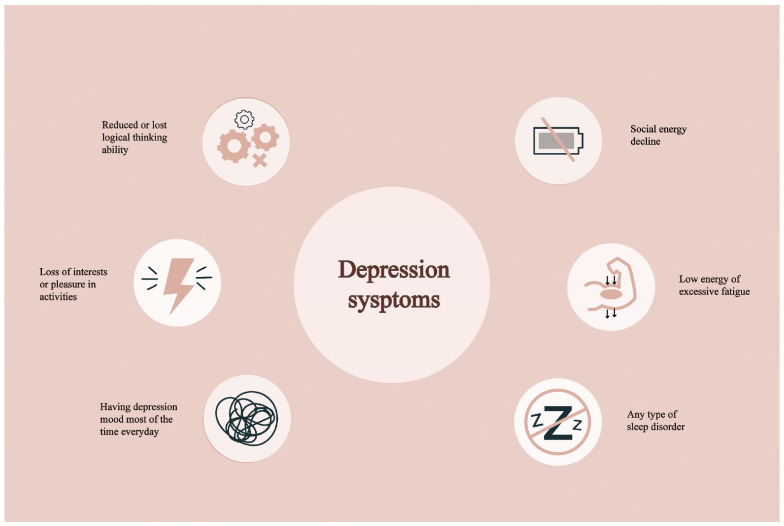
The typical symptoms of depression.

Depression is a pervasive and debilitating mental health condition that affects over 300 million individuals worldwide annually [1]. Its symptoms, including fatigue, diminished motivation, insomnia, and a profound sense of hopelessness, are all too common in everyday life (Figure 1) [2]. According to the World Health Organization (WHO) in 2023, an estimated 3.8% of the global population, including 5% of adults, experiences depression. Among adults, the prevalence is 4% among men and 6% among women, indicating that depression is approximately 50% more prevalent in women than in men [3]. Among individuals over the age of 60, the prevalence rises to 5.7% [4]. Overall, approximately 280 million people worldwide suffer from depression. Notably, more than 10% of pregnant women and those in the postpartum period are also affected [5]. It is predicted that depression may soon become the leading cause of the global disease burden [6].

The etiology and mechanisms of depression are complex and multifaceted, involving genetic, biological, and sociopsychological dimensions. Genetic factors play a significant role in the onset of depression, with research indicating a certain degree of familial aggregation [7]. Specifically, individuals with a family history of depression may harbor genetic variations or susceptibility genes in their genetic material that are associated with the development of depression. These genetic factors have the potential to influence the structure and function of the brain during an individual’s development, as well as in their interaction with the environment, significantly increasing the risk of developing depression. In addition, biological factors play a crucial role in the onset of depression, particularly changes in brain biology and neurochemistry. The imbalance of neurotransmitters in the brain is one of the important physiological bases for the development of depression. Specifically, serotonin, as a key neurotransmitter, is widely involved in regulating mood, appetite, sleep, and cognitive function [8]. When serotonin levels decrease or its function is impaired, it may lead to low mood, appetite disturbances, and sleep disorders, which are core symptoms of depression. Dopamine is closely related to the reward system, motivation, and emotional control, and its imbalance may result in a lack of pleasure, decreased motivation, and emotional instability, thereby triggering or exacerbating depression. Norepinephrine, on the other hand, affects alertness, stress response, and emotional state, and abnormalities in its levels or function may also contribute to the emergence of depressive symptoms. Therefore, the imbalance of neurotransmitters such as serotonin, dopamine, and norepinephrine plays a pivotal role in the pathogenesis of depression. Furthermore, sociopsychological factors also play a crucial role in the pathogenesis of depression. Long-term or acute psychosocial stress can induce or exacerbate depressive symptoms through various pathways. Major negative life events, such as the loss of a loved one or unemployment, can serve as triggering factors, activating the individual’s stress response system [9]. This leads to sustained hyperactivity of the hypothalamic-pituitary-adrenal axis and elevated cortisol levels, which in turn impair hippocampal neurogenesis and affect prefrontal cortex function. Chronic social stress and early traumatic experiences may induce negative cognitive patterns, forming a predisposing psychological state for depression and increasing susceptibility to depression in adulthood. These factors interact with one another, collectively determining the onset and progression of the disease.

To address depression, numerous therapies have been developed, and the efficacy of antidepressants has been estimated. For instance, tricyclic antidepressants, a classic type of antidepressant medication, are effective for some patients; however, they are associated with toxicity to the central nervous system and cardiovascular system [10]. Recent research has found that complexing these drugs with β-cyclodextrin or its derivatives can significantly enhance their solubility and stability, reduce systemic toxicity, improve pharmacokinetics, and decrease adverse reactions [11]. Another research has found that the combined use of ketamine with aripiprazole can effectively mitigate ketamine’s dissociative effects without compromising its antidepressant efficacy, thereby enhancing patients’ treatment experience and comfort levels [12]. In a rat model of depression, an intelligent transdermal drug delivery system based on thermosensitive hydrogel effectively controlled drug release dosage through electrothermal heating. By employing a liposomal formulation, this system significantly improved the percutaneous permeability of selegiline, resulting in notable improvements in depressive symptoms. Furthermore, it reduced the levels of pro-inflammatory cytokines in the serum and mitigated hippocampal damage [13]. Currently, considerable research has been conducted on the biomechanisms underlying depression, leading to the establishment of well-established guidelines for the use of antidepressants. The primary pharmacological approach to treating depression involves modulating neurotransmission [14]. However, two-thirds of patients with major depressive disorder do not respond to initial treatment options. Furthermore, roughly 30% of patients fail to respond to at least two standard antidepressants at the prescribed dose and duration of six weeks or more, a condition known as treatment-resistant depression [15]. This unresponsiveness is influenced by various factors, including age route of administration, metabolic factors, pharmacodynamics of the drug and so on. In light of this scenario, there is a pressing need for the development of new antidepressants or innovative dosage forms to address treatment resistance in these patients.

In this review, we delve into the pivotal factors that hinder the full efficacy of antidepressants and present novel strategies to overcome these obstacles, including the discovery of novel antidepressants and the development of advanced dosage forms. We place particular emphasis on the utilization of polymers in drug delivery systems, an aspect frequently neglected in current literature. By synthesizing these approaches, we aim to provide valuable insights and inspire future progress in enhancing the therapeutic potency of antidepressants.

## 2. Causes of Depression

The onset of mental disorders, such as depression, results from a complex interplay of genetic, biological, psychological, social, and environmental factors, with particular emphasis on the interaction between genetic factors and the environment [16]. These factors also play a crucial role in determining the severity and individual variability of depression.

### 2.1. Environmental Risk Factors

Environmental factors that influence depression are diverse, ranging from the neighborhood environment, life and academic stress [17,18,19], disability, poorer life satisfaction, and medical comorbidities [20] to disease or physiological factors such as human immunodeficiency virus (HIV), cancer, post-pregnancy issues, changes in the gut microbiome, and others [21,22,23,24], and so on. In recent years, these two sorts of factors have also been combined in the analysis. For example, a study revealed the joint effects of ambient air pollution exposure and genetic susceptibility on depression and anxiety. Subsequently, genome-wide by environment interaction studies (GWEISs) were conducted to evaluate the interaction effects of genetic variants and air pollution on the risk of depression and anxiety [25].

### 2.2. Hormonal Imbalance Factors

Numerous clinical studies have demonstrated that individuals suffering from endocrine disorders and chronic systemic diseases exhibit a significantly elevated risk of developing depression [26,27,28], highlighting the crucial contribution of biological imbalances to the pathogenesis of depression. A cross-sectional study has revealed that women using hormonal contraceptives exhibit a higher prevalence of depression, and this association is linked to the type of contraceptive used and the duration of its usage [29]. It is particularly noteworthy that postpartum depressive symptoms are significantly associated with certain measures of cortisol, while stress is unrelated to any cortisol indicators. Anxiety and depression may exhibit different and even opposite characteristics of cortisol dysregulation [30]. Furthermore, a study found that in euthyroid individuals with mild to moderate depression, there exists a significant positive correlation between sensitivity to thyroid hormones and sleep duration, with the Thyroid-Stimulating Hormone Index (TSHI) playing a particularly important role in reflecting this association [31]. Follicle-stimulating hormone (FSH) is capable of inducing depression-like behaviors in mice, and this induction is closely associated with neuroinflammation, impairment of synaptic plasticity, and disruption of the glutamate/gamma-aminobutyric acid (GABA) cycle triggered by FSH, suggesting that FSH may play a significant role in the pathogenesis of depression [32].

### 2.3. Genome-Wide Association Studies (GWAS)

Genomic alterations and inheritance significantly increase the risk of depression [33,34,35]. In recent years, large-scale GWAS have provided significant insights into the genetic underpinnings of depression. The study identified genetic loci significantly associated with depression, encompassing known neurotransmitter-related genes, synaptic plasticity genes, and neuroinflammatory pathway genes. Through polygenic risk score analysis, these loci collectively accounted for the genetic risk of depression. The research further revealed significant genetic correlations between depression and anxiety disorders, as well as bipolar disorder, and identified multiple shared risk loci across these psychiatric disorders. These findings provide crucial insights into the molecular mechanisms of depression and pave the way for the development of precise predictive tools [36]. Furthermore, studies have shown an association between the short allele of the 5-HTTLPR gene and anxiety-related traits, marking an important step in understanding the relationship between 5-HTTLPR gene variations and mood disorders, including depression [37]. Moreover, GWAS have led to the identification of multiple genetic loci that exhibit significant associations with depression, including the gene regions of OLFM4 and NEGR1 [38]. These findings provide crucial guidance for personalized antidepressant treatment, aiding doctors in selecting more suitable medications and treatment plans based on patients’ genetic profiles.

## 3. Mechanisms of Depression

**Figure 2:**
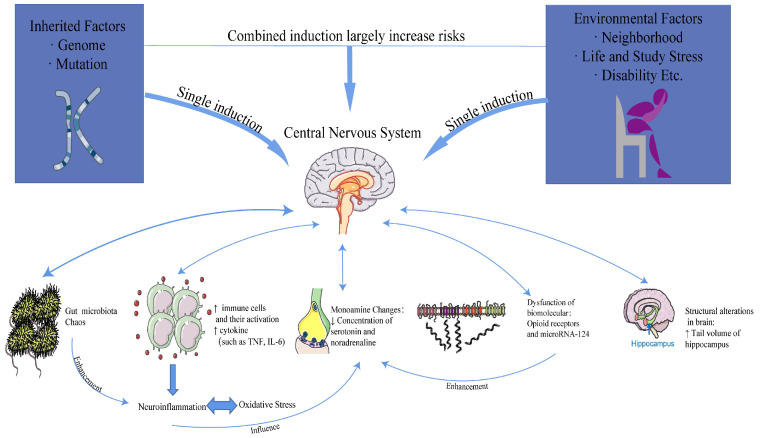
The relationship between causes and mechanisms.

Mounting evidence supports the underlying mechanisms of depression, which encompass alterations in monoamine neurotransmitters and inflammation, structural changes and damage to the brain, alterations in the gut microbiota, and oxidative stress. The relationship between causes and mechanisms is illustrated in Figure 2.

### 3.1. Monoamine Neurotransmitter Changes

Monoamines, such as serotonin, dopamine, and catecholamines (including adrenaline and noradrenaline), are thought to play a role in stress-induced physiological responses. It is postulated that stress initiates a cascade of these events, which may alter the neurodevelopmental trajectories of brain structure, function, and connectivity, ultimately contributing to the onset of psychiatric disorder [39]. In the 1950s, the discovery that other drugs could modify the bioavailability of catecholamines led to the initial formulation of the monoamine hypothesis [40].

### 3.2. Inflammation

Inflammation, orchestrated by the immune system, is primarily induced by both external and internal stressors, leading to a multitude of diseases, including psychiatric conditions. Depression exhibits a strong correlation with inflammation, marked by elevated levels of oxidative stress and nitric oxide. These factors, through an impaired immune response, fuel the inflammatory process and intensify the progression of depression [41]. As research delves deeper, the understanding of a cytokine network implicated in the pathology of depression continues to evolve [42], paving the way for future studies to substantiate the neuroinflammation hypothesis.

### 3.3. Neuroplasticity and Critical Periods

Growing evidence implicates impaired neuroplasticity as a core pathological feature of depression. Central to this process is the downregulation of BDNF/TrkB signaling, which leads to abnormal synaptic pruning and reduced expression of key synaptic proteins. These molecular changes correlate with structural and functional alterations in depression, particularly weakened connectivity between the prefrontal cortex and limbic system, as observed in neuroimaging studies [43,44]. 

### 3.4. Gut-Brain Axis Dysregulation

The gut-brain axis plays a crucial role in depression, with gut microbiota dysbiosis and microbial metabolites influencing neuroinflammation and behavior. Chronic stress reduces beneficial bacteria while increasing pro-inflammatory taxa, altering short-chain fatty acid (SCFA) production. Notably, butyrate levels decline in the brain under stress, correlating with microglial hyperactivation and impaired neurogenesis. Rifaximin, a gut-targeted antibiotic, restores microbial balance, elevates brain butyrate, and shifts microglia toward an anti-inflammatory state, mitigating depressive-like behaviors. These findings highlight SCFAs as key mediators linking gut microbiota to microglial function and synaptic plasticity in depression [45].

### 3.5. Oxidative Stress and Mitochondrial Dysfunction

Gut microbiota dysbiosis exacerbates oxidative stress by reducing SCFAs such as butyrate. As a histone deacetylase inhibitor, butyrate enhances mitochondrial biogenesis and reduces ROS production. Its deficiency impairs the function of mitochondrial complexes I/III, leading to increased electron leakage from the electron transport chain and accumulation of superoxide anions (O_2_^−^). Concurrently, elevated lipopolysaccharide activates NADPH oxidase through the TLR4/NF-κB pathway, further generating reactive oxygen species and creating a vicious cycle of oxidative stress and mitochondrial damage. This mechanistic link explains why interventions with probiotics or butyrate supplementation can simultaneously improve mitochondrial function and oxidative stress markers [46].

## 4. Factors Influencing the Efficacy of Antidepressants

The limited effectiveness of antidepressants poses a significant challenge in the treatment of depression, as two-thirds of patients with major depressive disorder fail to respond to first-line treatments, and there is a high rate of recurrence. The degree of insufficient therapeutic effects is affected by various factors, including drug factors and body factors.

### 4.1. Drug Factors

Oral administration remains the most convenient and widely adopted method for delivering antidepressants. Tablets and capsules, as two prevalent dosage forms for oral antidepressant therapy, offer the advantage of easy daily dosage tracking [47]. They are also recognized as the safest and most well-tolerated options, even for pediatric prescriptions [48,49,50,51,52]. Moreover, capsules have proven to be a vital carrier for herbal antidepressants [53,54]. Nevertheless, efforts are underway to enhance the consistent therapeutic performance of these dosage forms. For instance, reducing particle size (such as through micronization and ultramicronization) can enhance solubility and bioavailability. Reducing the particle size increases the surface area, thereby promoting dissolution and improving absorption. However, the pharmacokinetic parameters of antidepressants of these two dosage forms still fall short of ideal levels [55,56], which in turn limits their antidepressant effects [57]. This can be attributed to factors such as the first-pass effect and the blood-brain barrier.

Additionally, the lipophilicity of antidepressants, as indicated by the Log P parameter, influences their absorption and distribution, with first-line antidepressants often exhibiting significant distribution issues. These issues can be exacerbated by age, particularly in the elderly [58].

### 4.2. Body Factors

**Figure 3:**
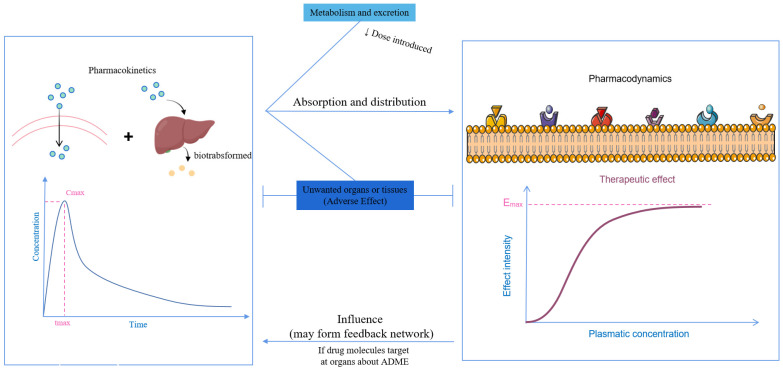
The relationship between pharmacokinetics and pharmacodynamics.

When considering the pharmacokinetics that influence the effect of antidepressants, metabolism and distribution are the two primary factors that determine the utilization of administered antidepressants. The body presents numerous barriers, stemming from both anatomical features and the physiochemical properties of the antidepressants themselves. Furthermore, pharmacokinetics serves as the foundation for the intensity of the pharmacodynamic response, and they can form a feedback network that mutually affects each other (Figure 3).

The first-pass effect is a prevalent metabolic process that significantly diminishes the oral dosage of medications, necessitating adjustments to ensure therapeutic levels within the bloodstream. Numerous orally administered antidepressants, including duloxetine and ketamine, are known to undergo metabolism that impacts their therapeutic efficacy [59,60]. The metabolism is mainly conducted by cytochrome P450, a highly polymorphic enzyme family that plays a crucial role in first-pass metabolism, thereby reducing the overall bioavailability. Notably, CYP2C19 is a vital cytochrome involved in the metabolism of many antidepressants, such as fluoxetine, venlafaxine, and vortioxetine [61].

The blood-brain barrier (BBB) serves as the primary defense mechanism for the brain, effectively blocking the entry of pathogens or harmful proteins from the plasma and maintaining the homeostasis of the brain environment. However, this barrier also poses a challenge for the delivery of antidepressants to the brain [62]. This is largely due to P-glycoprotein, a prominent type of efflux transporter located in the epithelial cells of brain blood vessels. P-glycoprotein significantly reduces the bioavailability of antidepressants by actively expelling them from the cells as they attempt to cross the BBB [63]. It is of much importance to consider the drug-drug interaction and drug-food interactions.

**Figure 4:**
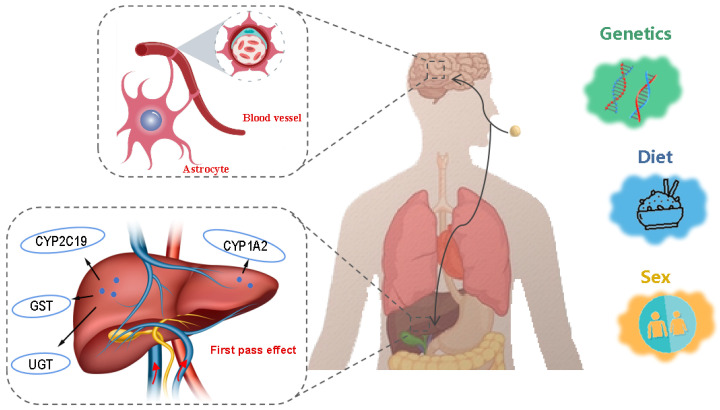
Body factors influencing the efficacy of antidepressants. Images from https://freepik.com and https://vecteezy.com (accessed on 8 February 2025).

Inter-individual genetic variability, sex, age, and diet have been demonstrated to exert great influences on the pharmacokinetics of antidepressants (Figure 4), as the metabolized activity of enzymes such as liver drug enzymes and the tight degree of the blood-brain barrier are closely related to these factors [64,65]. Consequently, the administration of the same antidepressant can result in substantial differences in efficacy among individuals. These variations among patients pose a considerable challenge to the development of effective personalized antidepressant therapies [66].

### 4.3. Common Factors Between Drugs and the Body

Genetic polymorphisms in CYP450 enzymes, particularly CYP2D6 and CYP2C19, significantly influence antidepressant treatment outcomes by altering drug metabolism kinetics. Clinical evidence demonstrates that CYP2C19 poor metabolizers exhibit 2.6-fold higher escitalopram plasma concentrations compared to normal metabolizers, substantially increasing the risk of adverse effects including QT prolongation, while CYP2D6 ultrarapid metabolizers show markedly enhanced venlafaxine metabolism that may compromise therapeutic efficacy due to insufficient active metabolite formation. Pharmacogenomic-guided treatment strategies effectively address these variations: dose reduction (50% for escitalopram in CYP2C19 poor metabolizers) and alternative drug selection (e.g., non-CYP2D6 substrates such as mirtazapine for ultrarapid metabolizers) have been shown to improve treatment response rates by 30–50% while significantly reducing adverse events [61,67,68].

## 5. Solutions for Improving Depression

As a consequence of the aforementioned factors, a wide array of solutions has emerged, highlighting the diversity and effectiveness of antidepressant treatments. Among these, antidepressant medications hold a significant position. Table 1 presented their acting point, effects, and representative drugs. Furthermore, the introduction of new dosage forms has broadened the range of choices for antidepressant therapy.

**Table 1:** Classification, acting point, effect, and representative drugs of antidepressants.

Classification	Acting Point	Effect	Efficacy	Efficacy Rate	Side Effects	Representative Drugs	Refs.
Selective serotonin reuptake inhibitors (SSRIs)	Serotonin Transport Protein	↑ Concentration of serotonin	50–60% response rate in major depressive disorder (MDD) (HAM-D reduction ≥ 50%)	50–60% (MDD)	Withdrawal syndrome, such as the flu, feeling sleepy	Fluoxetine, paroxetine, sertraline, fluvoxamine, escitalopram, and citalopram	[69,70,71]
Serotonin-noradrenaline reuptake inhibitors (SNRIs)	Serotonin Transport Protein, Norepinephrine Transport Protein	↑ Concentration of serotonin and noradrenaline in a balance	55–65% response rate, may work better in severe depression	50–65% (MDD)	Withdrawal syndrome, such as flu-like feelings, sleepiness, and granulocytosis (very rare)	Venlafaxine, desvenlafaxine, duloxetine, milnacipran, and levomilnacipran	[72,73,74]
Serotonin Receptor Antagonists and Reuptake Inhibitors (SARIs)	Serotonin Transport Protein, 5-HT2 Receptor	↑ Concentration of serotonin ↑ Concentration of noradrenaline ↓ Production of hydrogen peroxide	45–55% response rate, rapid sedation	45–55% (MDD)	Drowsiness, priapism (rare but serious)	Trazodone and nefazodone	[75,76,77]
Norepinephrine and dopamine reuptake inhibitors (NDRIs)	Norepinephrine Transport Protein, Dopamine Transport Protein	↑ Concentration of norepinephrine and dopamine	50–60% response rate, may improve fatigue and cognition	45–55% (MDD)	Seizure risk, insomnia	Bupropion	[78,79,80]
Melatonin receptor agonists and 5-HT2C receptor antagonists	Melatonin Receptor, 5-HT2C Receptor	↑ Concentration of serotonin ↑ Concentration of dopamine ↓ Production of hydrogen peroxide	40–50% response rate, improves sleep architecture	40–50% (MDD, insomnia)	Liver monitoring required.	Agomelatine	[81,82]
NMDA receptor antagonist	NMDA Receptor	↑ Release of glutamine, leading to the activation of subtype glutamate receptors	70% rapid response (within 24 h) in TRD, effects last ~1 week	60–70% (TRD)	Hypertension	Ketamine	[83,84,85,86,87,88,89]

↑ indicates an increase in the concentration or content of a substance, while ↓ indicates a decrease in the concentration or content of a substance.

### 5.1. New Antidepressants

#### 5.1.1. Selective Serotonin Reuptake Inhibitors (SSRIs)

Fluoxetine, commonly referred to as Prozac, exhibits anti-inflammatory and neuroprotective properties in addition to its serotonergic effects [90]. When combined with low-dose clonazepam, it can expedite the treatment process for patients suffering from depression while also minimizing side effects [91]. The efficacy and tolerability of citalopram are influenced by variations in CYP2C19 activity [92,93]. Furthermore, the concomitant use of these medications with aspirin may elevate the risk of bleeding complications [94]. In a randomized clinical trial, escitalopram was found to significantly reduce the depression scores of patients with mild to moderate depression following coronary artery bypass grafting surgery. After eight weeks of treatment, it also markedly improved the patients’ quality of life. Notably, no significant difference in drug side effects was observed between escitalopram and the placebo group [95].

#### 5.1.2. Serotonin and Norepinephrine Reuptake Inhibitors (SNRIs)

In a meta-analysis, venlafaxine was demonstrated to be an effective monotherapy option for patients with depression, significantly alleviating both anxiety and depressive symptoms [96]. However, its use is associated with certain side effects, including sexual dysfunction and overactive bladder [97]. Recent studies have suggested that combining venlafaxine with mirtazapine can enhance its antidepressant efficacy while mitigating adverse effects on sexual function [98]. Duloxetine, another SNRI has been shown to not only improve depressive symptoms but also reduce serum concentrations of pro-inflammatory cytokines such as IL-8, IL-12, and IFN-γ, highlighting its potential anti-inflammatory properties [99]. In a clinical trial involving 2598 patients, levomilnacipran ER demonstrated significant improvements in noradrenergic and anxiety symptoms compared to placebo, enhancing response rates and exhibiting notable enhancement in the total Sheehan Disability Scale score. These improvements were primarily mediated by the alleviation of noradrenergic symptoms. This finding contributes to our understanding of the mechanism of action of levomilnacipran ER in the treatment of MDD and provides a valuable reference for clinical practice [100]. In a 10-month open-label extension study, evaluated using the 17-item Hamilton Depression Rating Scale, the results indicated that among subjects who did not respond to double-blind placebo, venlafaxine ER, and desvenlafaxine, 48% to 67% of patients, respectively, responded to open-label desvenlafaxine. For those who responded to the double-blind treatments, over 80% maintained their response to open-label desvenlafaxine. Additionally, desvenlafaxine was found to be well-tolerated. Overall, desvenlafaxine demonstrated favorable efficacy and tolerability in patients with major depressive disorder [101].

#### 5.1.3. Serotonin Receptor Antagonists and Reuptake Inhibitors (SARIs)

In the personalized treatment of depression, trazodone has been shown to enhance sleep quality and alleviate depressive symptoms, proving particularly effective for patients suffering from insomnia [102]. The combination of trazodone with hypothalamic phospholipids not only ameliorates symptoms associated with severe depression but also mitigates side effects such as increased heart rate linked to trazodone, thereby enhancing overall safety [103]. Nefazodone represents another novel agent within the class of SARIs. Research indicates that the co-administration of nefazodone and trazodone operates through traditional mechanisms involving monoamine reuptake inhibition while also influencing serotonin transporter function via allosteric regulation and pharmacological synergism. This dual action offers a promising new therapeutic strategy for managing depression [104].

#### 5.1.4. Norepinephrine and Dopamine Reuptake Inhibitors (NDRIs)

Unlike other antidepressants, bupropion lacks serotonergic activity [80]. When combined with dextromethorphan, it has been proven to rapidly improve the quality of life for patients with depression [105]. Furthermore, combination therapy using bupropion alongside SSRIs or SNRIs has demonstrated greater efficacy and tolerability in managing depressive symptoms compared to monotherapy [106]. Additionally, bupropion has been found to possess anti-inflammatory properties [107].

#### 5.1.5. Melatonin Receptor Agonists and 5-HT2C Receptor Antagonists

Agomelatine modulates the sleep-wake cycle by activating MT1 and MT2 receptors while antagonizing 5-HT2C receptors. This mechanism enhances the release of dopamine and norepinephrine in the prefrontal cortex, thereby improving mood and sleep quality. Clinical studies have demonstrated that agomelatine is both effective and well-tolerated in patients experiencing depression accompanied by anxiety symptoms [108]. Furthermore, combined treatment with SSRIs, such as sertraline, may contribute to alleviating depressive symptoms and enhancing cognitive function in individuals, all within a high safety profile [47].

#### 5.1.6. Others

Ketamine was first synthesized in the US, and its antidepressant effect was discovered after various studies [83]. It is classified as a non-competitive antagonist of the N-methyl-D-aspartate receptor (NMDAR), which regulates calcium influx [84]. This mechanism underlies its analgesic and anesthetic properties [85,86]. Ketamine disrupts glutamatergic neurotransmission by inhibiting the NMDAR through downstream α2 adrenergic receptors and gamma-aminobutyric acid B (GABAB) receptors [109]. Additionally, it enhances the release of glutamine, leading to the activation of subtype glutamate receptors located in the postsynaptic membrane of the medial prefrontal cortex, thereby alleviating depressive symptoms in mice [110]. Research has also shown that different enantiomers of ketamine exhibit varying antidepressive potencies, with the order of (R)-ketamine > (R, S)-ketamine > (S)-ketamine [111]. However, prolonged use or abuse of ketamine can lead to addiction [89]. The euphoria and pleasure induced by ketamine may lead users to develop psychological dependency, ultimately resulting in addictive behavior. Abuse of ketamine can cause an imbalance of neurotransmitters in the brain, particularly affecting neurotransmitters such as glutamate and γ-aminobutyric acid. This imbalance may further exacerbate addictive behaviors, leading to cognitive impairments, liver and kidney dysfunction, and even schizophrenia-like symptoms in users. In severe cases, it can be life-threatening.

Probiotics are microorganisms that have been proven to stimulate and regulate the proliferation of gastrointestinal microorganisms, benefiting human health [112]. The representative probiotics used in medical applications include Lactobacillus and Bifidobacterium. Research has shown that commensal bacteria can influence the levels of neurotransmitters in the brain, such as serotonin and dopamine, which play pivotal roles in regulating emotions and behavior. Furthermore, by modulating the composition of the gut microbiota, particularly by increasing the number and diversity of commensal bacteria, it is possible to alleviate mental health issues such as anxiety and depression [113]. In multiple clinical trials included in the analysis, the scores on depression scales for the probiotic group were generally lower than those for the placebo group. This improvement was observed across patients of different ages, genders, and depression severity levels, indicating that probiotics contribute to alleviating depressive symptoms. While the overall results support the positive effects of probiotics on depression, there was significant heterogeneity among the studies. This may be related to factors such as the type of probiotics, dosage, intervention duration, and individual differences among the study subjects [114].

Vortioxetine, as a multimodal antidepressant, has shown remarkable improvement in depressive symptoms among patients in the results of a meta-analysis, with statistically significant differences compared to the placebo group. Similarly, based on testing with the Digit Symbol Substitution Test, an assessment tool for cognitive function, the vortioxetine-treated group outperformed the placebo group, indicating that vortioxetine can enhance cognitive function in patients with MDD. These findings provide robust evidence for the use of vortioxetine in the treatment of MDD [115].

Desipramine belongs to the class of tricyclic antidepressants, and a recent study has indicated that chronic desipramine treatment can reverse deficits in cell activity, norepinephrine innervation, and anxiety-depression phenotypes in a fluoxetine-resistant cF1ko mouse model. This reveals the potential efficacy of chronic desipramine treatment in fluoxetine-resistant depression, providing new insights and directions for the treatment of depression [116]. Nortriptyline is another tricyclic antidepressant (TCA). Research has revealed that, in a six-month, double-blind, randomized clinical trial, the average depression scores before treatment were comparable between the nortriptyline group and the fluoxetine group, with scores of 32.85 ± 6.23 and 33.12 ± 6.50, respectively. Following treatment, the score change in the nortriptyline group was 13.4 ± 4.68, whereas in the fluoxetine group, it was 16.96 ± 4.96. The results indicated that both nortriptyline and fluoxetine were effective in treating major depressive disorder; however, fluoxetine demonstrated superior efficacy [117].

Vilazodone is a selective serotonin reuptake inhibitor and a partial 5-HT1A receptor agonist. In a randomized, double-blind, placebo-controlled trial, it was found that patients in the vilazodone treatment group showed significant improvement in depressive symptoms, as measured by changes in standardized depression rating scales such as the Hamilton Depression Rating Scale, with statistically significant differences compared to the placebo group. Furthermore, vilazodone demonstrated a favorable safety profile in the trial, with most adverse reactions being mild to moderate in severity and well-tolerated by patients [118].

Recent studies have established TrkB as a critical mediator of antidepressant effects through BDNF signaling. Rapid-acting agents such as ketamine directly bind TrkB receptors as molecular wedges, stabilizing their active conformation and rapidly enhancing synaptic plasticity independent of BDNF synthesis [85,119]. In contrast, conventional SSRIs/SNRIs require chronic administration to upregulate BDNF-TrkB signaling and promote neuroplastic changes [120,121]. The clinical relevance of this pathway is highlighted by the BDNF Val66Met polymorphism, which impairs TrkB activation and predicts poorer treatment response [122]. These findings suggest TrkB-targeted compounds may represent a promising new antidepressant class [123].

The application of artificial intelligence (AI) is becoming increasingly widespread in the field of drug discovery. Research indicates that by employing AI technologies such as deep learning and machine learning, it is possible to analyze vast amounts of biological data more efficiently and identify potential biomarkers and therapeutic targets associated with MDD, thereby expediting the development process of novel antidepressant medications and enabling personalized precision medicine [124]. In another study, AI approaches were utilized to examine metabolite data derived from the plasma and urine samples of 295 participants, aiming to unravel the metabolic connections between depression and chronic fatigue syndrome. The research uncovered several shared metabolic biomarkers at the metabolic level between these two disorders, which may potentially serve as novel biomarkers for the future development of antidepressant drugs [125]. However, its application also confronts numerous challenges. Firstly, data quality and accessibility serve as critical constraints. Privacy restrictions surrounding pharmaceutical development data have led to a scarcity of high-quality datasets, making it imperative to establish a unified mechanism for open data sharing to enrich the repositories available for drug research and development. Moreover, the multi-scale validation of AI-driven predictions is cost-intensive. From virtual screening to clinical trials, AI predictions necessitate iterative validation, with high experimental failure rates driving up R&D costs. Strategies such as ensemble learning can be leveraged in conjunction with multi-source data to enhance predictive accuracy.

### 5.2. New Dosage Form of Antidepressants

The development of appropriate dosage forms plays a pivotal role not only in facilitating the administration of medications but also in optimizing their pharmacokinetic profiles and dosing characteristics [126]. This principle equally applies to antidepressants, where the design of dosage forms can significantly enhance their therapeutic efficacy and patient compliance [127,128].

#### 5.2.1. Injection

Injection represents a highly crucial dosage form for the administration of antidepressants, primarily due to its capacity to enhance the bioavailability of the active ingredient significantly. As an illustration, a study conducted in China demonstrated that injectable levosulpiride achieved a quicker attainment of steady state and exhibited substantially superior bioavailability [129]. The administration of ketamine via injection has been proven to exhibit a swift therapeutic effect while being well-tolerated. To evaluate the synergistic effects of various substances in the treatment of depression, numerous compounds are injected into animal models. For example, experimental studies have shown that the combination of imipramine and citicoline in mice demonstrates an enhanced antidepressant effect [130]. Similarly, the pairing of geniposide with eleutheroside B has also been proven to yield beneficial outcomes in terms of alleviating depressive symptoms [131].

#### 5.2.2. Patch

The patch facilitates direct absorption of the drug into the bloodstream, enabling swift distribution throughout the body’s tissues and effective penetration of the BBB to target the central nervous system. This method circumvents the liver’s first-pass effect, consequently enhancing the drug’s bioavailability. For patients suffering from severe depression, transdermal administration of selegiline has proven efficacious while maintaining excellent tolerability and safety profiles [132,133]. Furthermore, contemporary research has revealed that selegiline microneedle array patches formulated with nanostructured lipid carriers exhibit an extended half-life and augmented brain bioavailability, marking a significant advancement in treatment options [134].

#### 5.2.3. Extended-Release/Controlled-Release Formulations

Sustained-release and controlled-release formulations play a crucial role in the treatment of depression. These preparations regulate the rate of drug release within the body, allowing for prolonged therapeutic effects that effectively alleviate symptoms of depression. Sustained-release ketamine tablets have been shown to enhance the rate of drug absorption, mitigate side effects such as elevated blood pressure and heart rate, and improve both the tolerance and safety profile of the medication. For example, R-107 exhibits prolonged therapeutic effects, with clinical trials reporting a 6.1-point reduction in MADRS scores versus placebo at 13 weeks and a relapse rate of 42.9%. However, their slower onset (≥4 weeks to peak efficacy) may limit utility in acute settings [135]. Sustained-release trazodone maintains a consistent concentration level of the drug in the body by steadily releasing it over time, thereby providing a prolonged and stable antidepressant effect [136]. Furthermore, controlled-release paroxetine has effectively alleviated clinical symptoms in patients with depression while reducing the incidence of adverse events [137].

#### 5.2.4. Nasal Spray

The BBB tightly regulates the transport of various molecules, thereby preventing antidepressants from accessing the central nervous system [62]. Nasal administration offers a unique advantage in bypassing both the BBB and first-pass metabolism, leading to improved bioavailability [138]. The intranasal delivery of esketamine demonstrates a pharmacokinetic advantage over oral administration, with absolute bioavailability reaching 48% (versus 8% for oral esketamine) and a significantly reduced time to peak plasma concentration (T_max_ = 0.5–1 h vs. 5–6 h for oral tablets) [139,140]. Furthermore, antidepressants formulated as nanosized particles, emulsions, or even liposomes can be effectively delivered through intranasal delivery systems, enhancing their potential for therapeutic application [141]. When desvenlafaxine is delivered via the nose-to-brain route using mucoadhesive PLGA-chitosan nanoparticles, the intranasally administered optimized desvenlafaxine-loaded nanoparticles significantly alleviate depressive symptoms, elevate monoamine levels in the brain, and enhance the pharmacokinetic profile of the drug in the brains of rodents compared to oral administration [142]. Delivery of selegiline hydrochloride via the intranasal route using thiolated chitosan nanoparticles (TCNs) exhibits significantly higher concentrations of selegiline hydrochloride in the brain compared to oral administration, resulting in more effective alleviation of depressive symptoms. This underscores the potential application of TCNs in the treatment of depression [143]. However, clinical experience has shown that prolonged use can lead to tolerability issues, ranging from mild nasal irritation to more severe epithelial damage. The withdrawal of zinc-containing nasal sprays due to anosmia serves as a stark reminder of the fragility of olfactory tissues. Even with newer formulations such as esketamine, where rigorous clinical trials have confirmed the preservation of olfactory function, transient symptoms such as nasal congestion and postnasal drip are still frequently reported by patients. These findings highlight the importance of meticulous formulation design that balances therapeutic efficacy with local tissue compatibility [141,144].

#### 5.2.5. Orally Disintegrating Tablets

Orally dissolving tablets, also known as orally disintegrating tablets, allow patients to take their medication without the need for water, as they rapidly dissolve in the mouth. This formulation is especially advantageous for individuals who have difficulty swallowing or are resistant to taking conventional tablet forms. Mirtazapine oral disintegrating tablets offer convenience, which enhances adherence among patients with depression, and they are well-tolerated and preferred by patients as a treatment option. While orally disintegrating tablets offer practical advantages, their pharmacokinetic parameters (e.g., bioavailability, T_max_) remain comparable to conventional oral formulations, as confirmed in bioavailability studies [52,145].

#### 5.2.6. Nanoparticle-Based Formulations

Liposomes, composed of phospholipids, represent a relatively uncommon dosage form for antidepressants in clinical practice. However, they have demonstrated the capability to enhance drug potency and improve bioavailability by increasing the solubility of the drug [146,147]. Liposomes facilitate the delivery of antidepressants across the BBB to reach active sites in the brain [148]. Additionally, liposomes exhibit reduced toxicity and low immunogenicity, which can prolong retention time and optimize drug release characteristics [149,150]. In the early 1980s, researchers observed that liposomes enhanced the antidepressant effects in mice, reducing depression-like behaviors [151]. More recently, a 2020 study described liposome-based nanocarriers of eugenol, which exhibited superior antianxiety effects through better blockade of the neurokinin-1 (NK-1) receptor, which also regulates depressive processes. This experiment suggests the potential for increased antidepressant efficacy when delivered via liposomes [152]. Furthermore, liposomes can detoxify overdosed antidepressants by transferring their ingredients from the cardiovascular system to fat, muscle, and skin [153]. However, the rapid elimination of liposomes by the reticuloendothelial system limits their clinical use in antidepressant therapy, necessitating appropriate modifications [154]. Despite these challenges, liposomes continue to hold promise as a delivery system for antidepressants due to their unique advantages in enhancing drug delivery and efficacy.

Polymers have proven to be exceptionally effective in enhancing bioavailability when incorporated into drug delivery systems. They exhibit exceptional efficiency in modifying pharmacokinetic properties, allowing for the adjustment of pharmacokinetic parameters to achieve desired therapeutic outcomes. By combining these polymers with existing dosage forms, significant improvements in pharmaceutical effects can be achieved. For example, polymers can prolong drug release time and mitigate potentially high transient plasma concentrations, effectively reducing C_max_. This indicates their potential for use in future finished drug products. Notably, polymers exhibit high compatibility and can be seamlessly integrated with a wide range of dosage forms, from conventional tablets to advanced liposomes, as outlined in Table 2. Given their versatility and effectiveness, the use of polymers in the market as a delivery vehicle for antidepressants is highly recommended for the future. Their ability to tailor pharmacokinetic properties and enhance bioavailability makes them a valuable addition to the pharmaceutical toolkit.

Furthermore, polymer-mediated antidepressant therapy has demonstrated promising avenues for enhancing therapeutic efficacy through various formulation strategies. Researchers have loaded hypericin (HYP) onto black phosphorus nanosheets (BP) modified with the neural cell-targeting peptide RVG29, synthesizing a nanoplatform named BP-RVG29@HYP (BRH). BRH can effectively traverse an in vitro BBB model and significantly alleviate depressive-like behaviors and oxidative stress in mice. Furthermore, BRH exhibits excellent safety with minimal side effects [155]. The latest research has developed an inflammation-targeting, microglia-biomimetic nanodelivery system (PDA-Mem@M), which leverages the homotypic affinity of microglial cell membranes to achieve targeted delivery to inflammatory cells. Through the synergistic effects of polydopamine and memantine, this system demonstrates better therapeutic efficacy and biosafety for depression [156]. Another study has found that transferrin-modified carboxymethyl chitosan-chitosan nanoparticles can specifically bind to transferrin receptors on the BBB, enabling efficient transmigration across the BBB through receptor-mediated transcytosis. In the neuroinflammatory and neuronal injury environments associated with depression, the permeability of the BBB increases, further enhancing the BBB targeting efficiency of these nanoparticles. Additionally, the nanoparticles exhibit good safety and biocompatibility in both in vitro and in vivo experiments, providing new strategies and hope for the treatment of depression [157]. The H@EFCP, another type of self-healing hydrogel dressing, is composed of components such as carboxymethyl chitosan, polyvinyl alcohol, and Prussian blue nanoparticles. This dressing facilitates the healing of burn wounds by modulating the inflammatory microenvironment. Furthermore, it mitigates the intensification of central inflammation triggered by peripheral oxidative stress, consequently lowering the incidence of depression linked to central inflammation [158]. When employing chitosan spray-dried microcapsules as a controlled-release delivery system for venlafaxine hydrochloride, optimized ratios of chitosan to sodium tripolyphosphate can achieve controlled release of venlafaxine, enhancing the effectiveness of the treatment and improving patient compliance [159]. Using the O/W emulsion solvent evaporation method, long-acting injectable microspheres loaded with agomelatine were prepared. Pharmacokinetic studies have demonstrated that these microspheres can release the drug at a stable plasma concentration for up to 30 days in vivo, making them a promising carrier for the treatment of major depressive disorder [160]. These advancements address key challenges in depression treatment while potentially reducing the latency period for therapeutic effects.

**Table 2:** Application of polymers in delivery.

Polymers	Forms	Loaded Drug	Functions	Results	Ref.
Polylactic acid	Coating of nanospheres	Venlafaxine	Controlled release	An obvious delay of release in intestinal fluid, with a prevention of burst in gastric fluid.	[161]
Poly(lactic-co-glycolic acid) (PLGA)	Microparticles	Mirtazapine	Controlled release	The release rate was very close to zero order, and extended release was achieved.	[162]
	Intranasally delivered nanoparticles	Agomelatine	Enhancement of effects	The antidepressant effect was improved, obviously.	[163]
Chitosan	Microsphere	Mirtazapine	Modification of pharmacokinetic properties	Clear improvements in PK parameters, including AUC, half-life, and reduced clearance, were shown.	[164]
	Intranasally delivered nanoparticles	Venlafaxine	Targeting delivery	Through comparison, the concentration of venlafaxine delivered by nanoparticles was much higher in the brain, especially the effect on the increase of brain/blood ratios and drug transport efficiency, showing its high efficiency.	[165]
	Nanoparticles (with modification of Tween 80)	Minocycline	Targeting delivery	The modified nanoparticles showed a better efficiency for target transporting and higher safety.	[166]
	Nanoparticles (with modification of Tween 80)	Gallic acid	Enhancement of effects, targeting delivery	Decreases in activity in monoamine oxidase and malondialdehyde levels were in expectation.	[167]
	Interpenetrating polyelectrolyte nanocomplexes (IPNC) (with pectin)	Citalopram	Controlled release (extended)	The in vitro, in vivo, and histopathological examinations all showed good drug effects and great extended properties, with the most extended gained when the complex is made of chitosan and pectin in a 3:1 ratio.	[168]
	Intranasally delivered thermoreversible biogel (with glycerophosphate)	Doxepin	Targeting delivery	The bioavailability was clearly improved, and prolonged release was achieved as expected.	[169]
Combination of polyoxyethylene (25) lauryl ether and β-cyclodextrin	Nasal spray particles	GLP-2 peptide	Improvement on CNS transitivity and pharmacodynamic effect	The stability of GLP-2 pep was increased, and the CNS migration profile was good, as well as the antidepressant effect.	[170]
polyethylene oxide and polysiloxane	hydrophilic-hydrophobic copolymer networks	Protriptyline	Controlled release	As the content of polyethylene oxide changes, the release rate of the drug can be controlled.	[171]
Polyethylcellulose	Outer coating networks (NE30D form another coating)	Venlafaxine	Controlled release	As the coatings were added, the drug release profile was satisfactory, and stability was good.	[172]
	Semi-interpenetrating hydrogels (with anionic polyamidoamine dendrimers)	Venlafaxine	Controlled release (extended)	The release rate is largely slowed by the addition of dendrimers, while the hydrogel behavior was affected by certain content of PEG.	[173]
Poloxamer	Self-assembled thermosensitive hydrogel (P407, P188, and alginate)	Icariin	Thermosensitive, controlled release	The release rate had a satisfactory zero-order kinetic property, and low doses had a fast and good antidepressant effect.	[174]
	Intranasally delivered thermoresponsive in situ gel (P407, P188)	Agomelatine	Targeting delivery	The bioavailability in the brain was largely increased, and the pharmacodynamic effects of agomelatine were increased largely.	[175]
		Berberine	Enhancement of effects	A better antidepressant effect was achieved, and a lower dose could be used.	[176]
Alginate	Intranasally delivered nanogel-based thermosensitive hydrogel	Albiflorin	Enhancement of effects	Higher bioavailability was approved, as a lower dose can have better antidepressant effects, and prolonged release was achieved.	[177]
PLGA and PC combination	Nanospheres	Duloxetine	Modification of pharmacokinetic properties	The brain concentration of duloxetine was increased three times compared with the oral solution.	[59]
Poly(ε-caprolactone)	Nanosphere capsules (With lipid core)	Trazodone	Controlled release	A controlled release rate was achieved no matter what kind of oil core it was, and the antidepressant effect was increased obviously.	[178]
Ethylene Vinyl Acetate	Films	Curcumin	Enhanced solubility and stability	Improved solubility and stability of curcumin in EVA films for potential pharmaceutical applications.	[179]
Poly(vinyl alcohol)	Nanocapsules	Paclitaxel	Targeted delivery and controlled release	Targeted delivery of paclitaxel to cancer cells with controlled release properties.	[180]
Poly(lactic acid)-PEG copolymer	Micelles	Doxorubicin	Targeted delivery and reduced toxicity	Effective targeting of cancer cells with reduced toxicity to normal tissues.	[181]
Chitosan-Alginate composite	Microcapsules	Probiotics	Enhanced stability and gut delivery	Improved stability of probiotics and enhanced delivery to the gut.	[182]
Poly(β-amino ester)	Nanoparticles	Vaccine antigen	Enhanced immune response	Improved immune response and antigen presentation.	[183]
Poly(styrene-co-maleic acid)	Nanofibers	Growth factors	Biocompatible, controlled release, tissue engineering support	Promoted tissue regeneration and healing.	[184]
Poly(ethylene oxide)	Micelles	Gene therapeutics	Enhanced gene transfection efficiency	Improved gene therapy outcomes with minimal side effects	[185]
Poly(N-isopropylacrylamide) (PNIPAM)	Thermosensitive Hydrogels	Proteins and Peptides	Temperature-responsive release	Controlled release based on temperature changes, cell protection.	[186]
Poly(glycolic acid)	Microspheres	Anti-inflammatory Drugs	Controlled release	Reduced inflammation and improved tissue healing.	[187]
Polyurethane	Films	Antibiotics	Antimicrobial activity, wound healing support	Reduced infection risk and accelerated wound healing.	[188]

## 6. Discussion

Depression, a pervasive mental health issue on a global scale, exerts profound impacts on individuals, families, and society alike. It is far more than just a “temporary low mood” or “bad day”; it is a chronic and severe mental condition that can rob patients of their interests, cause anhedonia, reduce energy levels, and even trigger suicidal thoughts. According to statistics from the World Health Organization, depression has emerged as a primary cause of disability worldwide, posing a significant challenge to public health. With the acceleration of life’s pace and the intensification of social pressures, the incidence of depression has been steadily rising year by year, and there is a noticeable trend of younger individuals being affected. This underscores the paramount importance of prevention and treatment efforts targeting depression.

In this review, we delve into the causes and mechanisms of depression and the factors influencing the efficacy of antidepressant medications and propose solutions for improving depression. Depression, as a complex mental illness, exhibits a diverse range of causes, pathogenic mechanisms, and factors impacting treatment outcomes, necessitating a multi-faceted understanding and response.

Firstly, the causes and mechanisms of depression encompass biological, psychological, and social dimensions. From a biological perspective, neurotransmitter imbalance, particularly involving serotonin, dopamine, and norepinephrine, is widely recognized as one of the core biological mechanisms underlying the onset of depression. These neurotransmitters play pivotal roles in regulating mood, cognition, and behavior, and their imbalance can directly lead to mood dysregulation. Additionally, systemic inflammation as well as localized inflammatory responses in the brain are considered closely linked to the initiation and progression of depression. Meanwhile, structural changes and damage to the brain, such as reductions in hippocampal volume, constitute an essential component of the biological mechanisms of depression. In recent years, with the deepening of research on the gut microbiota, there has been a growing recognition that dysbiosis of the gut microbiota may also influence brain function and emotional states through the “gut-brain axis,” thus emerging as a novel biological factor in the pathogenesis of depression. Psychologically, early life experiences exert profound influences on an individual’s mental health. Furthermore, an individual’s coping strategies and personality traits are also deemed significant psychological factors influencing the development of depression. Socially, high levels of life stress, interpersonal relationship problems, and insufficient social support are equally non-negligible factors in the onset of depression.

The factors influencing the efficacy of antidepressant drugs are also highly complex and diverse. From the perspective of drug metabolism, the metabolic process of antidepressants in the human body is not straightforward but is primarily mediated by the CYP450 enzyme family. The CYP450 enzyme family is a group of mixed-function oxidase systems widely present in the liver and other tissues. They play a crucial role in drug metabolism by catalyzing the oxidation reactions of various drugs, thereby altering their chemical structures and pharmacological activities. Within this enzyme family, the CYP2C19 enzyme is of particular importance for the metabolism of antidepressants. CYP2C19 can specifically recognize and bind to antidepressant drug molecules, and through a series of biochemical reactions, convert the drugs into metabolites that are more easily excreted from the body. However, the polymorphism of the CYP2C19 enzyme can lead to variations in drug metabolism, which in turn affects the drug’s bioavailability and efficacy. In addition, individual differences among patients can also impact the efficacy of antidepressants. For example, patients of different age groups have variations in bodily functions and metabolic capacities. Men and women differ in hormone levels, body composition, and physiological functions. Other factors such as genetic background, comorbid conditions, and concomitant medications also come into play. Therefore, in clinical practice, these factors must be taken into account to optimize antidepressant treatment regimens.

In terms of solutions for improving depression, antidepressant medications occupy a prominent position. Different types of antidepressants possess distinct mechanisms of action and efficacy profiles and have demonstrated significant advantages in alleviating depressive symptoms. However, the resistance and effectiveness of antidepressants pose a significant challenge in therapy. A report points out that one-third of patients had no response to conventional antidepressants and still had the risks of self-harm, even for onset patients, some of whom were not observed to have a relief of symptoms of MDD in a short period [189]. Another clinical trial points out that antidepressants only had a 54% response rate, meaning a considerable number of patients cannot achieve full recovery through medication [190]. Furthermore, most antidepressants require continuous administration for weeks or even months before their therapeutic effects gradually become apparent, which can be a challenge for patients with acute depression. During the waiting period for the medication to take effect, patients may experience prolonged pain and discomfort. This, in turn, can lead to poor patient adherence and may even cause patients to discontinue treatment. The first-pass effect and blood-brain barrier present major obstacles in reducing the bioavailability of antidepressants and slowing down the onset. Moreover, the integrity of the BBB and the regulation of related proteins are crucial components in understanding the pathogenesis of depression, providing a theoretical basis for the development of new therapeutic approaches [191,192].

Therefore, research on antidepressants targeting bypassing the first-pass effect and the blood-brain barrier holds great promise for the future. To date, the outcomes of these experiments have been promising, with the emergence of novel pharmacological targets for rapid-acting antidepressants such as ketamine [189] and the use of ultrasound to open the BBB, enhancing hippocampal neurogenesis and improving the efficacy of antidepressants. Furthermore, the exploration of pharmaceutical compounds that can penetrate the BBB barrier remains a focal point of research [193,194]. The relationship between depression and other diseases may also offer a new way of thinking antidepressant design, which may target the crossing receptor of two diseases [195]. The anatomical and microcosmic structures of the brain and their changes are also significant references for antidepressant design [196,197].

It is noteworthy that in recent years, the application of polymers in antidepressant delivery systems has garnered considerable attention. By devising appropriate polymer carriers, targeted drug delivery and controlled release can be achieved, thus boosting the efficacy of antidepressants and minimizing side effects. For instance, polymers such as PLGA (poly(lactic-co-glycolic acid)) and chitosan have been widely employed in the fabrication of nanoparticles, microspheres, liposomes, and other delivery systems for the administration of antidepressants. These delivery systems not only improve the brain-targeting efficacy of the drugs but also prolong their duration of action through controlled-release mechanisms, ultimately enhancing patient compliance. These advancements provide a vast platform for the application of novel dosage forms in the industrial production of antidepressants in the future [198]. In addition, the long-term safety of the polymer is very important. This can be achieved through molecular design strategies—such as incorporating hydrolytic/oxidative-resistant functional groups—and the addition of anti-aging agents (e.g., UV absorbers, antioxidants), in conjunction with accelerated aging experiments, to predict performance degradation under specific environmental conditions (e.g., elevated temperatures, humidity). Simultaneously, the development of degradable polymers and the establishment of closed-loop recycling systems can mitigate the risk of microplastic accumulation. A pivotal approach involves optimizing material selection via life cycle assessment (LCA) to ensure compliance with FDA standards in medical applications, ultimately achieving a harmonious balance between safety and sustainability.

On the ethical front, personalized medicine utilizes genetic data and clinical information to tailor treatment plans for individuals, a process that must strictly adhere to data protection and privacy regulations to ensure the security of patient information. Meanwhile, before receiving personalized medical treatment and novel therapies, patients should be fully informed of relevant details, including potential risks and benefits, so as to make autonomous decisions. For the ethical evaluation of novel therapies, in addition to focusing on their scientific efficacy and safety, factors such as economic burden, social equity, and accessibility must also be taken into account. For instance, exorbitant treatment costs may prevent some patients from accessing necessary care, thereby sparking ethical controversies.

## 7. Conclusions

This review delves into the etiology and pathogenesis of depression and the challenges faced in its treatment, with a particular focus on the innovative applications of polymers in antidepressant drug delivery systems. Depression is a complex disorder with multifactorial origins, where treatment response is intricately influenced by a web of genetic, environmental, physiological, and pharmacological factors. Key factors contributing to the suboptimal efficacy of antidepressant therapies include metabolic enzyme polymorphisms, drug interactions, and the permeability of the BBB. The development of novel antidepressant drugs has offered new avenues for the treatment of depression. Concurrently, polymers, as a vital component of drug delivery systems, have demonstrated immense potential in enhancing drug efficacy and improving targeting specificity. Future research should further concentrate on optimizing the design of polymeric delivery systems and formulating personalized treatment strategies based on individual differences. Meanwhile, while promoting the development of personalized medicine and novel therapies, it is imperative to establish a robust ethical review mechanism to ensure that medical innovations conform to ethical standards and safeguard the rights and safety of patients.

## Data Availability

Not applicable.

## Abbreviations

The following abbreviations are used in this manuscript:

HIV	Human immunodeficiency virus
GWEIS	Genome-wide by environment interaction studies
SSRIs	Selective serotonin reuptake inhibitors
SNRIs	Serotonin-noradrenaline reuptake inhibitors
SARIs	Serotonin Receptor Antagonists and Reuptake Inhibitors
NDRIs	Norepinephrine and dopamine reuptake inhibitors
NMDAR	N-methyl-D-aspartate receptor
GABAB	Gamma-aminobutyric acid B
MDD	Major depressive disorder
GWAS	Genome-Wide Association Studies
BBB	Blood-brain barrier
NK-1	Neurokinin-1
PLGA	Polylactic-co-glycolic acid
IPNC	Interpenetrating polyelectrolyte nano-complexes
SJW	St John’s Wort
FSH	Follicle-stimulating hormone
TSHI	Thyroid-Stimulating Hormone Index
GABA	Glutamate/gamma-aminobutyric acid
FMNs	Fluorescein-loaded magnetic nanoparticles
Tf	Transferrin
HYP	Hypericin
BP	Black phosphorus
BRH	BP-RVG29@HYP
SCFA	Short-chain fatty acid
TCNs	Thiolated chitosan nanoparticles
TCA	Tricyclic antidepressant
AI	Artificial intelligence
LCA	Life cycle assessment

## References

[1] Pu D., Luo J., Wang Y., Ju B., Lv X., Fan P., He L. (2018). Prevalence of depression and anxiety in rheumatoid arthritis patients and their associations with serum vitamin D level. Clin. Rheumatol..

[2] Rakel R.E. (1999). Depression. Prim. Care.

[3] Bromet E., Andrade L.H., Hwang I., Sampson N.A., Alonso J., de Girolamo G., de Graaf R., Demyttenaere K., Hu C., Iwata N. (2011). Cross-national epidemiology of DSM-IV major depressive episode. BMC Med..

[4] Reed P.G. (1989). Mental health of older adults. West. J. Nurs. Res..

[5] Howard L.M., Molyneaux E., Dennis C.-L., Rochat T., Stein A., Milgrom J. (2014). Perinatal mental health 1 Non-psychotic mental disorders in the perinatal period. Lancet.

[6] Malhi G.S., Mann J.J. (2018). Depression. Lancet.

[7] Tabrizi F., Rosen J., Gronvall H., William-Olsson V.R., Arner E., Magnusson P.K., Palm C., Larsson H., Viktorin A., Bernhardsson J. (2025). Heritability and polygenic load for comorbid anxiety and depression. Transl. Psychiatry.

[8] Kanova M., Kohout P. (2021). Serotonin-Its Synthesis and Roles in the Healthy and the Critically Ill. Int. J. Mol. Sci..

[9] Grossi G., Ahs A., Lundberg U. (1998). Psychological correlates of salivary cortisol secretion among unemployed men and women. Integr. Physiol. Behav. Sci..

[10] Preskorn S.H., Fast G.A. (1991). Therapeutic drug monitoring for antidepressants: Efficacy, safety, and cost effectiveness. J. Clin. Psychiatry.

[11] Varut R.M., Popescu A.I.S., Gaman S., Niculescu C.E., Niculescu A.S., Dop D., Stepan M.D., Ionovici N., Singer C.E., Popescu C. (2025). Cyclodextrin-Based Drug Delivery Systems for Depression: Improving Antidepressant Bioavailability and Targeted Central Nervous System Delivery. Pharmaceutics.

[12] Nakatsuka D., Suwa T., Deguchi Y., Fujita Y., Tashima R., Ohnami S., Kawashima H., Oishi N., Ogawa K., Yamakawa H. (2025). Fine-tuning of dopamine receptor signaling with aripiprazole counteracts ketamine’s dissociative action, but not its antidepressant effect. Transl. Psychiatry.

[13] Liu S., Huang S., Liu K., Han Y., Xiong F. (2023). The novel design of an intelligent anti-depression transdermal drug delivery system. Biomaterials.

[14] Puryear C.B., Brooks J., Tan L., Smith K., Li Y., Cunningham J., Todtenkopf M.S., Dean R.L., Sanchez C. (2020). Opioid receptor modulation of neural circuits in depression: What can be learned from preclinical data?. Neurosci. Biobehav. Rev..

[15] Krishnan V., Nestler E.J. (2008). The molecular neurobiology of depression. Nature.

[16] Penner-Goeke S., Binder E.B. (2019). Epigenetics and depression. Dialogues Clin. Neurosci..

[17] Rajbhandari P., Shrestha D. (2018). Prevalence and Its Associated Risk Factors in Tooth Wear. J. Nepal Med. Assoc..

[18] Lun K.W., Chan C.K., Ip P.K., Ma S.Y., Tsai W.W., Wong C.S., Wong C.H., Wong T.W., Yan D. (2018). Depression and anxiety among university students in Hong Kong. Hong Kong Med. J..

[19] Quinn M.E., Stanton C.H., Slavich G.M., Joormann J. (2020). Executive Control, Cytokine Reactivity to Social Stress, and Depressive Symptoms: Testing the Social Signal Transduction Theory of Depression. Stress.

[20] Subramaniam M., Abdin E., Sambasivam R., Vaingankar J.A., Picco L., Pang S., Seow E., Chua B.Y., Magadi H., Mahendran R. (2016). Prevalence of Depression among Older Adults-Results from the Well-being of the Singapore Elderly Study. Ann. Acad. Med. Singap..

[21] Patten S.B. (2020). Current perspectives on co-morbid depression and multiple sclerosis. Expert Rev. Neurother..

[22] Kim B.S., Chung P.W., Kim B.K., Lee M.J., Park J.W., Chu M.K., Ahn J.Y., Bae D.W., Song T.J., Sohn J.H. (2020). The impact of remission and coexisting migraine on anxiety and depression in cluster headache. J. Headache Pain.

[23] Denton A.R., Samaranayake S.A., Kirchner K.N., Roscoe R.F., Berger S.N., Harrod S.B., Mactutus C.F., Hashemi P., Booze R.M. (2019). Selective monoaminergic and histaminergic circuit dysregulation following long-term HIV-1 protein exposure. J. Neurovirol..

[24] Winter G., Hart R.A., Charlesworth R.P.G., Sharpley C.F. (2018). Gut microbiome and depression: What we know and what we need to know. Rev. Neurosci..

[25] Zhao M., Chen L., Yang J., Han D., Fang D., Qiu X., Yang X., Qiao Z., Ma J., Wang L. (2018). BDNF Val66Met polymorphism, life stress and depression: A meta-analysis of gene-environment interaction. J. Affect. Disord..

[26] Gold P.W., Chrousos G.P. (1999). The endocrinology of melancholic and atypical depression: Relation to neurocircuitry and somatic consequences. Proc. Assoc. Am. Physicians.

[27] Bauer M., Heinz A., Whybrow P.C. (2002). Thyroid hormones, serotonin and mood: Of synergy and significance in the adult brain. Mol. Psychiatry.

[28] Dantzer R., O’Connor J.C., Freund G.G., Johnson R.W., Kelley K.W. (2008). From inflammation to sickness and depression: When the immune system subjugates the brain. Nat. Rev. Neurosci..

[29] Yusuf A.M., Warsame M.O., Gedi S., Abdullahi N.A., Ahmed D.I. (2024). Prevalence of Depression Among Women Using Hormonal Contraceptives in Mogadishu, Somalia: A Cross-Sectional Study. Open Access J. Contracept..

[30] Weiss S.J., Xu L. (2024). Postpartum symptoms of anxiety, depression and stress: Differential relationships to women’s cortisol profiles. Arch. Womens Ment. Health.

[31] Xiao X.Q., Fu F.S., Xiang C., Yan H.C. (2024). Sensitivity to thyroid hormones is associated with sleep duration in the euthyroid population with depression degree lower than moderate. Sci. Rep..

[32] Huang L.Q., Sun S.Q., Jiang G.G., Xie G.F., Yang Y.Y., Chen S.C., Luo J.Y., Lv C., Li X., Liao J.M. (2024). Follicle-stimulating hormone induces depression-like phenotype by affecting synaptic function. Front. Mol. Neurosci..

[33] Polderman T.J., Benyamin B., de Leeuw C.A., Sullivan P.F., van Bochoven A., Visscher P.M., Posthuma D. (2015). Meta-analysis of the heritability of human traits based on fifty years of twin studies. Nat. Genet..

[34] Geschwind D.H., Flint J. (2015). Genetics and genomics of psychiatric disease. Science.

[35] Buch A.M., Liston C. (2021). Dissecting diagnostic heterogeneity in depression by integrating neuroimaging and genetics. Neuropsychopharmacology.

[36] Als T.D.D., Kurki M.I.I., Grove J., Voloudakis G., Therrien K., Tasanko E., Nielsen T.T., Naamanka J., Veerapen K., Levey D.F.F. (2023). Depression pathophysiology, risk prediction of recurrence and comorbid psychiatric disorders using genome-wide analyses. Nat. Med..

[37] Lesch K.P., Bengel D., Heils A., Sabol S.Z., Greenberg B.D., Petri S., Benjamin J., Muller C.R., Hamer D.H., Murphy D.L. (1996). Association of anxiety-related traits with a polymorphism in the serotonin transporter gene regulatory region. Science.

[38] Maul S., Giegling I., Fabbri C., Corponi F., Serretti A., Rujescu D. (2020). Genetics of resilience: Implications from genome-wide association studies and candidate genes of the stress response system in posttraumatic stress disorder and depression. Am. J. Med. Genet. B Neuropsychiatr. Genet..

[39] Heitmann H., Andlauer T.F.M., Korn T., Muhlau M., Henningsen P., Hemmer B., Ploner M. (2020). Fatigue, depression, and pain in multiple sclerosis: How neuroinflammation translates into dysfunctional reward processing and anhedonic symptoms. Mult. Scler..

[40] Perez-Caballero L., Torres-Sanchez S., Romero-Lopez-Alberca C., Gonzalez-Saiz F., Mico J.A., Berrocoso E. (2019). Monoaminergic system and depression. Cell Tissue Res..

[41] Catena-Dell’Osso M., Bellantuono C., Consoli G., Baroni S., Rotella F., Marazziti D. (2011). Inflammatory and neurodegenerative pathways in depression: A new avenue for antidepressant development?. Curr. Med. Chem..

[42] Petralia M.C., Mazzon E., Fagone P., Basile M.S., Lenzo V., Quattropani M.C., Di Nuovo S., Bendtzen K., Nicoletti F. (2020). The cytokine network in the pathogenesis of major depressive disorder. Close to translation?. Autoimmun. Rev..

[43] Yang P., Chen H., Wang T., Su H., Li J., He Y., Su S. (2023). Electroacupuncture promotes synaptic plasticity in rats with chronic inflammatory pain-related depression by upregulating BDNF/TrkB/CREB signaling pathway. Brain Behav..

[44] Wu Y., Zhu Z., Lan T., Li S., Li Y., Wang C., Feng Y., Mao X., Yu S. (2023). Levomilnacipran Improves Lipopolysaccharide-Induced Dysregulation of Synaptic Plasticity and Depression-Like Behaviors via Activating BDNF/TrkB Mediated PI3K/Akt/mTOR Signaling Pathway. Mol. Neurobiol..

[45] Li H., Xiang Y., Zhu Z., Wang W., Jiang Z., Zhao M., Cheng S., Pan F., Liu D., Ho R.C.M. (2021). Rifaximin-mediated gut microbiota regulation modulates the function of microglia and protects against CUMS-induced depression-like behaviors in adolescent rat. J. Neuroinflamm..

[46] Jomova K., Raptova R., Alomar S.Y., Alwasel S.H., Nepovimova E., Kuca K., Valko M. (2023). Reactive oxygen species, toxicity, oxidative stress, and antioxidants: Chronic diseases and aging. Arch. Toxicol..

[47] Chen R., Liu X. (2024). Efficacy of Sertraline Combined with Agomelatine in the Treatment of Depression and its Influence on Cognitive Function. Indian J. Pharm. Sci..

[48] Kolyvanov G.B., Zherdev V.P., Gribakina O.G., Bochkov P.O., Shevchenko R.V., Litvin A.A., Blynskaya E.V., Bueva V.V. (2019). Comparative Preclinical Pharmacokinetics and Bioavailability of Antidepressant GSB-106 Tablet Form. Bull. Exp. Biol. Med..

[49] Hamer A.M., Hartung D.M., Haxby D.G., Ketchum K.L., Pollack D.A. (2006). Initial results of the use of prescription order change forms to achieve dose form optimization (consolidation and tablet splitting) of SSRI antidepressants in a state Medicaid program. J. Manag. Care Pharm. JMCP.

[50] Wagstaff A.J., Goa K.L. (2001). Once-weekly fluoxetine. Drugs.

[51] Davey C.G., Chanen A.M., Hetrick S.E., Cotton S.M., Ratheesh A., Amminger G.P., Koutsogiannis J., Phelan M., Mullen E., Harrison B.J. (2019). The addition of fluoxetine to cognitive behavioural therapy for youth depression (YoDA-C): A randomised, double-blind, placebo-controlled, multicentre clinical trial. Lancet Psychiatry.

[52] Danileviciute V., Sveikata A., Adomaitiene V., Gumbrevicius G., Fokas V., Sveikatiene R. (2009). Efficacy, tolerability, and preference of mirtazapine orally disintegrating tablets in depressed patients: A 17-week naturalistic study in Lithuania. Medicina.

[53] Sun X., Zhu F., Zhou J., Chang X., Li L., Hu H., Wang Z., Xiao W. (2018). Anti-migraine and anti-depression activities of Tianshu capsule by mediating Monoamine oxidase. Biomed. Pharmacother..

[54] Wu J., Zhang T., Yu L., Huang S., Yang Y., Yu S., Li J., Cao Y., Wei Z., Li X. (2019). Zhile Capsule Exerts Antidepressant-Like Effects through Upregulation of the BDNF Signaling Pathway and Neuroprotection. Int. J. Mol. Sci..

[55] Nasser A., Gomeni R., Wang Z., Kosheleff A.R., Xie L., Adeojo L.W., Schwabe S. (2021). Population Pharmacokinetics of Viloxazine Extended-Release Capsules in Pediatric Subjects With Attention Deficit/Hyperactivity Disorder. J. Clin. Pharmacol..

[56] Kees F., Jehkul A., Bucher M., Mair G., Kiermaier J., Grobecker H. (2003). Bioavailability of opipramol from a film-coated tablet, a sugar-coated tablet and an aqueous solution in healthy volunteers. Arzneim. Forsch..

[57] Wyska E. (2019). Pharmacokinetic considerations for current state-of-the-art antidepressants. Expert Opin. Drug Metab. Toxicol..

[58] Klotz U. (2009). Pharmacokinetics and drug metabolism in the elderly. Drug Metab. Rev..

[59] Singh G., Sarwal A., Sharma S., Prasad P., Kuhad A., Ali W. (2021). Polymer-based prolonged-release nanoformulation of duloxetine: Fabrication, characterization and neuropharmacological assessments. Drug Dev. Ind. Pharm..

[60] Glue P., Russell B., Medlicott N.J. (2021). Influence of formulation and route of administration on ketamine’s safety and tolerability: Systematic review. Eur. J. Clin. Pharmacol..

[61] Milosavljevic F., Bukvic N., Pavlovic Z., Miljevic C., Pesic V., Molden E., Ingelman-Sundberg M., Leucht S., Jukic M.M. (2021). Association of CYP2C19 and CYP2D6 Poor and Intermediate Metabolizer Status With Antidepressant and Antipsychotic Exposure: A Systematic Review and Meta-analysis. JAMA Psychiatry.

[62] Abbott N.J., Patabendige A.A., Dolman D.E., Yusof S.R., Begley D.J. (2010). Structure and function of the blood-brain barrier. Neurobiol. Dis..

[63] Schinkel A.H. (1999). P-Glycoprotein, a gatekeeper in the blood-brain barrier. Adv. Drug Deliv. Rev..

[64] Oz M.D., Ozdemir F., Suzen H.S. (2021). The Influence of Genetic Variations and Drug Interactions Based on Metabolism of Antidepressants and Anticonvulsants. Curr. Drug Metab..

[65] Ihezie S.A., Mathew I.E., McBride D.W., Dienel A., Blackburn S.L., Thankamani Pandit P.K. (2021). Epigenetics in blood-brain barrier disruption. Fluids Barriers CNS.

[66] Jha M.K., Trivedi M.H. (2018). Personalized Antidepressant Selection and Pathway to Novel Treatments: Clinical Utility of Targeting Inflammation. Int. J. Mol. Sci..

[67] Bousman C.A., Stevenson J.M., Ramsey L.B., Sangkuhl K., Hicks J.K., Strawn J.R., Singh A.B., Ruano G., Mueller D.J., Tsermpini E.E. (2023). Clinical Pharmacogenetics Implementation Consortium (CPIC) Guideline for *CYP2D6*, *CYP2C19*, *CYP2B6*, *SLC6A4*, and *HTR2A* Genotypes and Serotonin Reuptake Inhibitor Antidepressants. Clin. Pharmacol. Ther..

[68] Li D., Pain O., Chiara F., Wong W.L.E., Lo C.W.H., Ripke S., Cattaneo A., Souery D., Dernovsek M.Z., Henigsberg N. (2024). Metabolic activity of CYP2C19 and CYP2D6 on antidepressant response from 13 clinical studies using genotype imputation: A meta-analysis. Transl. Psychiatry.

[69] Cipriani A., Furukawa T.A., Salanti G., Geddes J.R., Higgins J.P., Churchill R., Watanabe N., Nakagawa A., Omori I.M., McGuire H. (2009). Comparative efficacy and acceptability of 12 new-generation antidepressants: A multiple-treatments meta-analysis. Lancet.

[70] Perez-Caballero L., Torres-Sanchez S., Bravo L., Mico J.A., Berrocoso E. (2014). Fluoxetine: A case history of its discovery and preclinical development. Expert Opin. Drug Discov..

[71] Lochmann D., Richardson T. (2019). Selective Serotonin Reuptake Inhibitors. Handb. Exp. Pharmacol..

[72] Shelton R.C. (2019). Serotonin and Norepinephrine Reuptake Inhibitors. Handb. Exp. Pharmacol..

[73] Bellantuono C., Vargas M., Mandarelli G., Nardi B., Martini M.G. (2015). The safety of serotonin-noradrenaline reuptake inhibitors (SNRIs) in pregnancy and breastfeeding: A comprehensive review. Hum. Psychopharmacol..

[74] Fava G.A., Benasi G., Lucente M., Offidani E., Cosci F., Guidi J. (2018). Withdrawal Symptoms after Serotonin-Noradrenaline Reuptake Inhibitor Discontinuation: Systematic Review. Psychother. Psychosom..

[75] Fagiolini A., Albert U., Ferrando L., Herman E., Muntean C., Palova E., Cattaneo A., Comandini A., Di Dato G., Di Loreto G. (2020). A randomized, double-blind study comparing the efficacy and safety of trazodone once-a-day and venlafaxine extended-release for the treatment of patients with major depressive disorder. Int. Clin. Psychopharmacol..

[76] Cuomo A., Bianchetti A., Cagnin A., De Berardis D., Di Fazio I., Incalzi R.A., Marra C., Neviani F., Laurenzi P.F., Nicoletti F. (2021). Trazodone: A multifunctional antidepressant. Evaluation of its properties and real-world use. J. Gerontol. Geriatr..

[77] Fagiolini A., Comandini A., Dell’Osso M.C., Kasper S. (2012). Rediscovering Trazodone for the Treatment of Major Depressive Disorder. CNS Drugs.

[78] Gurcan G., Gurcan A. (2021). Bupropion-induced leukopenia: A case report. Turk. J. Clin. Psychiatry.

[79] Kim J.L., Chercover D., Masoudi H., Foltz L. (2024). Bupropion-induced pancytopenia. BMJ Case Rep..

[80] Clark A., Tate B., Urban B., Schroeder R., Gennuso S., Ahmadzadeh S., McGregor D., Girma B., Shekoohi S., Kaye A.D. (2023). Bupropion Mediated Effects on Depression, Attention Deficit Hyperactivity Disorder, and Smoking Cessation. Health Psychol. Res..

[81] Dolder C.R., Nelson M., Snider M. (2008). Agomelatine Treatment of Major Depressive Disorder. Ann. Pharmacother..

[82] (2009). Agomelatine: New drug. Adverse effects and no proven efficacy. Prescrire Int..

[83] Pereira V.S., Hiroaki-Sato V.A. (2018). A brief history of antidepressant drug development: From tricyclics to beyond ketamine. Acta Neuropsychiatri..

[84] Sun Q., Cao W., Luo J. (2021). The roles of GluN3-containing N-methyl-D-aspartate receptor in central nerve system. J. Zhejiang Univ. Med. Sci..

[85] Zanos P., Moaddel R., Morris P.J., Riggs L.M., Highland J.N., Georgiou P., Pereira E.F.R., Albuquerque E.X., Thomas C.J., Zarate C.A. (2018). Ketamine and Ketamine Metabolite Pharmacology: Insights into Therapeutic Mechanisms. Pharmacol. Rev..

[86] Zarate C.A., Singh J.B., Carlson P.J., Brutsche N.E., Ameli R., Luckenbaugh D.A., Charney D.S., Manji H.K. (2006). A randomized trial of an N-methyl-D-aspartate antagonist in treatment-resistant major depression. Arch. Gen. Psychiatry.

[87] Trullas R., Skolnick P. (1990). Functional antagonists at the NMDA receptor complex exhibit antidepressant actions. Eur. J. Pharmacol..

[88] Ivan Ezquerra-Romano I., Lawn W., Krupitsky E., Morgan C.J.A. (2018). Ketamine for the treatment of addiction: Evidence and potential mechanisms. Neuropharmacology.

[89] Liu Y., Lin D., Wu B., Zhou W. (2016). Ketamine abuse potential and use disorder. Brain Res. Bull..

[90] Zhao Y., Shang P., Wang M., Xie M., Liu J. (2020). Neuroprotective Effects of Fluoxetine Against Chronic Stress-Induced Neural Inflammation and Apoptosis: Involvement of the p38 Activity. Front. Physiol..

[91] Londborg P.D., Smith W.T., Glaudin V., Painter J.R. (2000). Short-term cotherapy with clonazepam and fluoxetine: Anxiety, sleep disturbance and core symptoms of depression. J. Affect. Disord..

[92] Mrazek D.A., Biernacka J.M., O’Kane D.J., Black J.L., Cunningham J.M., Drews M.S., Snyder K.A., Stevens S.R., Rush A.J., Weinshilboum R.M. (2011). *CYP2C19* variation and citalopram response. Pharmacogenet. Genom..

[93] Mahajna M., Abu Fanne R., Berkovitch M., Tannous E., Vinker S., Green I., Matok I. (2023). Effect of CYP2C19 Pharmacogenetic Testing on Predicting Citalopram and Escitalopram Tolerability and Efficacy: A Retrospective, Longitudinal Cohort Study. Biomedicines.

[94] Chavez-Leon E., Ontiveros Uribe M.P., Gomez C.S. (2008). Selective serotonin reuptake inhibitors antidepressants (SSRIs). Salud Ment..

[95] Baradaran A., Ardakani M.R.K., Bateni F.S., Asadian-Koohestani F., Vahedi M., Aein A., Shahmansouri N., Sadighi G. (2024). The effect of escitalopram in treating mild to moderate depressive disorder and improving the quality of life in patients undergoing coronary artery bypass grafting—A double-blind randomized clinical trial. Front. Psychiatry.

[96] Rudolph R.L., Entsuah R., Chitra R. (1998). A meta-analysis of the effects of venlafaxine on anxiety associated with depression. J. Clin. Psychopharmacol..

[97] Klaas S., Siva J.B., Bak M., Govers M., Schreiber R. (2023). The pathophysiology of Post SSRI Sexual Dysfunction-Lessons from a case study. Biomed. Pharmacother..

[98] Alvarez-Silva A., Rodriguez-Manzo G., Reyes R., Fernandez-Guasti A. (2025). Combination of low doses of mirtazapine plus venlafaxine produces antidepressant-like effects in rats, without affecting male or female sexual behavior. Psychopharmacol..

[99] Gao W., Gao Y., Xu Y., Liang J., Sun Y., Zhang Y., Shan F., Ge J., Xia Q. (2024). Effect of duloxetine on changes in serum proinflammatory cytokine levels in patients with major depressive disorder. BMC Psychiatry.

[100] Blier P., Gommoll C., Chen C., Kramer K. (2017). Effects of levomilnacipran ER on noradrenergic symptoms, anxiety symptoms, and functional impairment in adults with major depressive disorder: Post hoc analysis of 5 clinical trials. J. Affect. Disord..

[101] Guico-Pabia C.J., Jiang Q., Ninan P.T., Thase M.E. (2011). Clinical outcomes following switch from venlafaxine ER to desvenlafaxine in nonresponders and responders. Curr. Med. Res. Opin..

[102] Incalzi R.A., Caraci F., Cuoml A., Fagiolini A., Strambi L.F. (2020). Personalized treatment of depression phenotypes: Role of trazodone in depression with insomnia. Riv. Psichiatr..

[103] Giannelli A., Rabboni M., Zarattini F., Malgeri C., Magnolfi G. (1989). A Combination of Hypothalamic Phospholipid Liposomes with Trazodone for Treatment of Depression—An Open Controlled-Study. Acta Psychiatry Scand..

[104] El-Kasaby A., Boytsov D., Kasture A., Krumpl G., Hummel T., Freissmuth M., Sandtner W. (2024). Allosteric Inhibition and Pharmacochaperoning of the Serotonin Transporter by the Antidepressant Drugs Trazodone and Nefazodone. Mol. Pharmacol..

[105] Willett K.C., Bond L.R., Morrill A.M., Lorena D., Petru I. (2024). Dextromethorphan/Bupropion: A Novel Treatment for Patients With Major Depressive Disorder. Am. J. Ther..

[106] Zisook S., Rush A.J., Haight B.R., Clines D.C., Rockett C.B. (2006). Use of bupropion in combination with serotonin reuptake inhibitors. Biol. Psychiatry.

[107] Yetkin D., Yilmaz I.A., Ayaz F. (2023). Anti-inflammatory activity of bupropion through immunomodulation of the macrophages. Naunyn-Schmiedebergs Arch. Pharmacol..

[108] Stein D.J. (2023). Evidence-Based Pharmacotherapy of Anxiety Symptoms in Patients with Major Depressive Disorder: Focus on Agomelatine. Neurol. Ther..

[109] Lur G., Fariborzi M., Higley M.J. (2019). Ketamine disrupts neuromodulatory control of glutamatergic synaptic transmission. PLoS ONE.

[110] Fukumoto K., Iijima M., Chaki S. (2016). The Antidepressant Effects of an mGlu2/3 Receptor Antagonist and Ketamine Require AMPA Receptor Stimulation in the mPFC and Subsequent Activation of the 5-HT Neurons in the DRN. Neuropsychopharmacology.

[111] Chang L., Zhang K., Pu Y., Qu Y., Wang S.M., Xiong Z., Ren Q., Dong C., Fujita Y., Hashimoto K. (2019). Comparison of antidepressant and side effects in mice after intranasal administration of (R,S)-ketamine, (R)-ketamine, and (S)-ketamine. Pharmacol. Biochem. Behav..

[112] Markowiak P., Śliżewska K. (2017). Effects of Probiotics, Prebiotics, and Synbiotics on Human Health. Nutrients.

[113] Foster J.A., Neufeld K.-A.M. (2013). Gut-brain: How the microbiome influences anxiety and depression. Trends Neurosci..

[114] Liu R.T., Walsh R.F.L., Sheehan A.E. (2019). Prebiotics and probiotics for depression and anxiety: A systematic review and meta-analysis of controlled clinical trials. Neurosci. Biobehav. Rev..

[115] McIntyre R.S., Harrison J., Loft H., Jacobson W., Olsen C.K. (2016). The Effects of Vortioxetine on Cognitive Function in Patients with Major Depressive Disorder: A Meta-Analysis of Three Randomized Controlled Trials. Int. J. Neuropsychopharmacol..

[116] Vahid-Ansari F., Zahrai A., Daigle M., Albert P.R. (2024). Chronic Desipramine Reverses De fi cits in Cell Activity, Norepinephrine Innervation, and Anxiety—Depression Phenotypes in Fluoxetine-Resistant cF1ko Mice. J. Neurosci..

[117] Hashemi S., Shirazi H.G., Mohammadi A., Zadeh-Bagheri G., Noorian K., Malekzadeh M. (2012). Nortriptyline versus fluoxetine in the treatment of major depressive disorder: A six-month, double-blind clinical trial. Clin. Pharmacol. Adv. Appl..

[118] Croft H.A., Pomara N., Gommoll C., Chen D., Nunez R., Mathews M. (2014). Efficacy and Safety of Vilazodone in Major Depressive Disorder: A Randomized, Double-Blind, Placebo-Controlled Trial. J. Clin. Psychiatry.

[119] Casarotto P.C., Girych M., Fred S.M., Kovaleva V., Moliner R., Enkavi G., Biojone C., Cannarozzo C., Sahu M.P., Kaurinkoski K. (2021). Antidepressant drugs act by directly binding to TRKB neurotrophin receptors. Cell.

[120] Castrén E., Monteggia L.M. (2021). Brain-Derived Neurotrophic Factor Signaling in Depression and Antidepressant Action. Biol. Psychiatry.

[121] Duman R.S., Aghajanian G.K., Sanacora G., Krysta J.H. (2016). Synaptic plasticity and depression: New insights from stress and rapid-acting antidepressants. Nat. Med..

[122] Notaras M., Du X., Gogos J., van den Buuse M., Hill R.A. (2017). The BDNF Val66Met polymorphism regulates glucocorticoid-induced corticohippocampal remodeling and behavioral despair. Transl. Psychiatry.

[123] Autry A.E., Monteggia L.M. (2012). Brain-Derived Neurotrophic Factor and Neuropsychiatric Disorders. Pharmacol. Rev..

[124] Stolfi F., Abreu H., Sinella R., Nembrini S., Centonze S., Landra V., Brasso C., Cappellano G., Rocca P., Chiocchetti A. (2024). Omics approaches open new horizons in major depressive disorder: From biomarkers to precision medicine. Front. Psychiatry.

[125] Zhang F.L., Wu C.H., Jia C.X., Gao K., Wang J.P., Zhao H.H., Wang W., Chen J.X. (2019). Artificial intelligence based discovery of the association between depression and chronic fatigue syndrome. J. Affect. Disord..

[126] Allen L.V. (2008). Dosage form design and development. Clin. Ther..

[127] Gibson K., Cartwright C., Read J. (2018). Conflict in Men’s Experiences With Antidepressants. Am. J. Mens Health.

[128] Frijlink H.W. (2003). Benefits of different drug formulations in psychopharmacology. Eur. Neuropsychopharmacol..

[129] Xu M., Zhou Y., Ni Y., He X., Li H., Sattar H., Chen H., Li W. (2015). Tolerability and Pharmacokinetic Comparison of Oral, Intramuscular, and Intravenous Administration of Levosulpiride After Single and Multiple Dosing in Healthy Chinese Volunteers. Clin. Ther..

[130] Khakpai F., Ramezanikhah M., Valizadegan F., Zarrindast M.R. (2021). Synergistic effect between imipramine and citicoline upon induction of analgesic and antidepressant effects in mice. Neurosci. Lett..

[131] Zhang B., Chang H.S., Hu K.L., Yu X., Li L.N., Xu X.Q. (2021). Combination of Geniposide and Eleutheroside B Exerts Antidepressant-like Effect on Lipopolysaccharide-Induced Depression Mice Model. Chin. J. Integr. Med..

[132] Bodkin J.A., Amsterdam J.D. (2002). Transdermal selegiline in major depression: A double-blind, placebo-controlled, parallel-group study in outpatients. Am. J. Psychiatry.

[133] Feiger A.D., Rickels K., Rynn M.A., Zimbroff D.L., Robinson D.S. (2006). Selegiline transdermal system for the treatment of major depressive disorder: An 8-week, double-blind, placebo-controlled, flexible-dose titration trial. J. Clin. Psychiatry.

[134] Patil A., Rajput A., Subbappa P., Pawar A. (2025). Formulation, development and in vivo characterization of selegiline hydrochloride nanostructured lipid nanocarrier loaded microneedle array patch for depression. Int. J. Pharm..

[135] Glue P., Medlicott N.J., Neehoff S., Surman P., Lam F., Hung N., Hung C.-t. (2020). Safety and efficacy of extended release ketamine tablets in patients with treatment-resistant depression and anxiety: Open label pilot study. Ther. Adv. Psychopharmacol..

[136] Di Nicola M., Pepe M., Panaccione I., Moccia L., Janiri L., Sani G. (2023). Update on Pharmacological Treatment for Comorbid Major Depressive and Alcohol Use Disorders: The Role of Extended-release Trazodone. Curr. Neuropharmacol..

[137] Golden R.N. (2003). Efficacy and tolerability of controlled-release paroxetine. Psychopharmacol. Bul..

[138] Kanojia G., Have R.T., Soema P.C., Frijlink H., Amorij J.P., Kersten G. (2017). Developments in the formulation and delivery of spray dried vaccines. Hum. Vacc. Immunother..

[139] Kryst J., Kawalec P., Pilc A. (2020). Efficacy and safety of intranasal esketamine for the treatment of major depressive disorder. Expert Opin. Pharmacoth..

[140] Molero P., Ramos-Quiroga J.A., Martin-Santos R., Calvo-Sánchez E., Gutiérrez-Rojas L., Meana J.J. (2018). Antidepressant Efficacy and Tolerability of Ketamine and Esketamine: A Critical Review. CNS Drugs.

[141] Xu J., Tao J., Wang J. (2020). Design and Application in Delivery System of Intranasal Antidepressants. Front. Bioeng. Biotechnol..

[142] Tong G.F., Qin N., Sun L.W. (2017). Development and evaluation of Desvenlafaxine loaded PLGA-chitosan nanoparticles for brain delivery. Saudi Pharm. J..

[143] Singh D., Rashid M., Hallan S.S., Mehra N.K., Prakash A., Mishra N. (2016). Pharmacological evaluation of nasal delivery of selegiline hydrochloride-loaded thiolated chitosan nanoparticles for the treatment of depression. Artif. Cells Nanomed. Biotechnol..

[144] Agrawal M., Saraf S., Saraf S., Antimisiaris S.G., Chougule M.B., Shoyele S.A., Alexander A. (2018). Nose-to-brain drug delivery: An update on clinical challenges and progress towards approval of anti-Alzheimer drugs. J. Control. Release.

[145] Ren H., Li T. (2014). Clinical applications of mirtazapine orally disintegrating tablets in depression treatment. Chin. J. New Drugs Clin. Remed..

[146] Nguyen T.X., Huang L., Gauthier M., Yang G., Wang Q. (2016). Recent advances in liposome surface modification for oral drug delivery. Nanomedicine.

[147] Lee Y., Thompson D.H. (2017). Stimuli-responsive liposomes for drug delivery. Wiley Interdiscip. Rev. Nanomed. Nanobiotechnol..

[148] Krauze M.T., Forsayeth J., Park J.W., Bankiewicz K.S. (2006). Real-time imaging and quantification of brain delivery of liposomes. Pharm. Res..

[149] Immordino M.L., Dosio F., Cattel L. (2006). Stealth liposomes: Review of the basic science, rationale, and clinical applications, existing and potential. Int. J. Nanomed..

[150] Ong J.C., Sun F., Chan E. (2011). Development of stealth liposome coencapsulating doxorubicin and fluoxetine. J. Liposome Res..

[151] Drago F., Continella G., Mason G.A., Hernandez D.E., Scapagnini U. (1985). Phospholipid liposomes potentiate the inhibitory effect of antidepressant drugs on immobility of rats in a despair test (constrained swim). Eur. J. Pharmacol..

[152] Siyal F.J., Memon Z., Siddiqui R.A., Aslam Z., Nisar U., Imad R., Shah M.R. (2020). Eugenol and liposome-based nanocarriers loaded with eugenol protect against anxiolytic disorder via down regulation of neurokinin-1 receptors in mice. Pak. J. Pharm. Sci..

[153] Howell B.A., Chauhan A. (2009). Binding of imipramine, dosulepin, and opipramol to liposomes for overdose treatment. J. Pharm. Sci..

[154] Ceh B., Lasic D.D. (1997). A Rigorous Theory of Remote Loading of Drugs into Liposomes: Transmembrane Potential and Induced pH-Gradient Loading and Leakage of Liposomes. J. Colloid Interf. Sci..

[155] Tan H., Cao K., Zhao Y., Zhong J., Deng D., Pan B., Zhang J., Zhang R., Wang Z., Chen T. (2024). Brain-Targeted Black Phosphorus-Based Nanotherapeutic Platform for Enhanced Hypericin Delivery in Depression. Small.

[156] Jiang C., Yang X., Huang Q., Lei T., Luo H., Wu D., Yang Z., Xu Y., Dou Y., Ma X. (2025). Microglial-Biomimetic Memantine-Loaded Polydopamine Nanomedicines for Alleviating Depression. Adv. Mater..

[157] He J.H., Yang L., Li D.M., Xie J.X., Zhou G.L., Zhou R.F., Li Y., Wei G.N., Gong Z.Q., Li L. (2025). Transferrin-modified carboxymethyl chitosan-chitosan nanoparticles as an efficient delivery carrier for targeted therapy of depression. Int. J. Biol. Macromol..

[158] Zhao W., Chen X., Han Z., Xun Z., Qi Y., Wang H., Chen C., Gong Z., Xue X. (2024). Nanoenzymes-Integrated and Microenvironment Self-Adaptive Hydrogel for the Healing of Burn Injury and Post-Burn Depression. Adv. Sci..

[159] Aranaz I., Panos I., Peniche C., Heras A., Acosta N. (2017). Chitosan Spray-Dried Microparticles for Controlled Delivery of Venlafaxine Hydrochloride. Molecules.

[160] Zhang H.J., Pu C.G., Wang Q., Tan X.Y., Gou J.X., He H.B., Zhang Y., Yin T., Wang Y.J., Tang X. (2019). Physicochemical Characterization and Pharmacokinetics of Agomelatine-Loaded PLGA Microspheres for Intramuscular Injection. Pharm. Res..

[161] Tang J., Slowing I.I., Huang Y., Trewyn B.G., Hu J., Liu H., Lin V.S. (2011). Poly(lactic acid)-coated mesoporous silica nanosphere for controlled release of venlafaxine. J. Colloid Interface Sci..

[162] Vysloužil J., Doležel P., Kejdušová M., Košťál V., Beneš L., Dvořáčková K. (2016). Long-term controlled release of PLGA microparticles containing antidepressant mirtazapine. Pharm. Dev. Technol..

[163] Jani P., Vanza J., Pandya N., Tandel H. (2019). Formulation of polymeric nanoparticles of antidepressant drug for intranasal delivery. Ther. Deliv..

[164] Ranjan O.P., Shavi G.V., Nayak U.Y., Arumugam K., Averineni R.K., Meka S.R., Sureshwar P. (2011). Controlled release chitosan microspheres of mirtazapine: In vitro and in vivo evaluation. Arch. Pharm. Res..

[165] Haque S., Md S., Fazil M., Kumar M., Sahni J.K., Ali J., Baboota S. (2012). Venlafaxine loaded chitosan NPs for brain targeting: Pharmacokinetic and pharmacodynamic evaluation. Carbohydr. Polym..

[166] Nagpal K., Singh S.K., Mishra D. (2013). Evaluation of safety and efficacy of brain targeted chitosan nanoparticles of minocycline. Int. J. Biol. Macromol..

[167] Nagpal K., Singh S.K., Mishra D.N. (2012). Nanoparticle mediated brain targeted delivery of gallic acid: In vivo behavioral and biochemical studies for improved antioxidant and antidepressant-like activity. Drug Deliv..

[168] Kamel R., Abbas H., El-Naa M. (2018). Composite carbohydrate interpenetrating polyelectrolyte nano-complexes (IPNC) as a controlled oral delivery system of citalopram HCl for pediatric use: In-vitro/in-vivo evaluation and histopathological examination. Drug Deliv. Transl. Res..

[169] Naik A., Nair H. (2014). Formulation and evaluation of thermosensitive biogels for nose to brain delivery of doxepin. BioMed Res. Int..

[170] Nakao Y., Horiguchi M., Nakamura R., Sasaki-Hamada S., Ozawa C., Funane T., Ozawa R., Oka J.I., Yamashita C. (2016). LARETH-25 and β-CD improve central transitivity and central pharmacological effect of the GLP-2 peptide. Int. J. Pharm..

[171] Sung C., Raeder J.E., Merrill E.W. (1990). Drug partitioning and release characteristics of tricyclic antidepressant drugs using a series of related hydrophilic-hydrophobic copolymers. J. Pharm. Sci..

[172] Guan T., Wang J., Li G., Tang X. (2011). Comparative study of the stability of venlafaxine hydrochloride sustained-release pellets prepared by double-polymer coatings and hot-melt subcoating combined with Eudragit(^®^) NE30D outercoating. Pharm. Dev. Technol..

[173] Yang H., Lopina S.T. (2005). Extended release of a novel antidepressant, venlafaxine, based on anionic polyamidoamine dendrimers and poly(ethylene glycol)-containing semi-interpenetrating networks. J. Biomed. Mater. Res. A.

[174] Xu D., Lu Y.R., Kou N., Hu M.J., Wang Q.S., Cui Y.L. (2020). Intranasal delivery of icariin via a nanogel-thermoresponsive hydrogel compound system to improve its antidepressant-like activity. Int. J. Pharm..

[175] Ahmed S., Gull A., Aqil M., Danish Ansari M., Sultana Y. (2019). Poloxamer-407 thickened lipid colloidal system of agomelatine for brain targeting: Characterization, brain pharmacokinetic study and behavioral study on Wistar rats. Colloids Surf. B..

[176] Wang Q.S., Li K., Gao L.N., Zhang Y., Lin K.M., Cui Y.L. (2020). Intranasal delivery of berberine via in situ thermoresponsive hydrogels with non-invasive therapy exhibits better antidepressant-like effects. Biomater. Sci..

[177] Xu D., Qiao T., Wang Y., Wang Q.S., Cui Y.L. (2021). Alginate nanogels-based thermosensitive hydrogel to improve antidepressant-like effects of albiflorin via intranasal delivery. Drug Deliv..

[178] Elhesaisy N., Swidan S. (2020). Trazodone Loaded Lipid Core Poly (ε-caprolactone) Nanocapsules: Development, Characterization and In Vivo Antidepressant Effect Evaluation. Sci. Rep..

[179] Han L., Yanling J., Jisheng Y., Rui P., Yanan C., Liuting M., Qiong J., Zhiyong Q. (2023). Preparation of curcumin-chitosan composite film with high antioxidant and antibacterial capacity: Improving the solubility of curcumin by encapsulation of biopolymers. Food Hydrocoll..

[180] Lee J.Y., Bae K.H., Kim J.S., Nam Y.S., Park T.G. (2011). Intracellular delivery of paclitaxel using oil-free, shell cross-linked HSA—Multi-armed PEG nanocapsules. Biomaterials.

[181] Yu J., Xie X., Xu X., Zhang L., Zhou X., Yu H., Wu P., Wang T., Che X., Hu Z. (2014). Development of dual ligand-targeted polymeric micelles as drug carriers for cancer therapy in vitro and in vivo. J. Mater. Chem. B.

[182] Malos I.G., Pasarin D., Ghizdareanu A.-I., Frunzareanu B. (2025). A Promising Approach for the Food Industry: Enhancing Probiotic Viability Through Microencapsulated Synbiotics. Microorganisms.

[183] Vartak A., Sucheck S.J. (2016). Recent Advances in Subunit Vaccine Carriers. Vaccines.

[184] Fan R., Zhang C., Li F., Li B., McCarthy A., Zhang Y., Chen S., Zhang L. (2024). Hierarchically Assembled Nanofiber Scaffolds with Dual Growth Factor Gradients Promote Skin Wound Healing Through Rapid Cell Recruitment. Adv. Sci..

[185] Su S., Tian Y., Li Y., Ding Y., Ji T., Wu M., Wu Y., Nie G. (2015). “Triple-Punch” Strategy for Triple Negative Breast Cancer Therapy with Minimized Drug Dosage and Improved Antitumor Efficacy. ACS Nano.

[186] Min Q., Yu X., Liu J., Wu J., Wan Y. (2019). Chitosan-Based Hydrogels Embedded with Hyaluronic Acid Complex Nanoparticles for Controlled Delivery of Bone Morphogenetic Protein-2. Pharmaceutics.

[187] Jeong C., Kim S.E., Shim K.-S., Kim H.-J., Song M.H., Park K., Song H.-R. (2018). Exploring the In Vivo Anti-Inflammatory Actions of Simvastatin-Loaded Porous Microspheres on Inflamed Tenocytes in a Collagenase-Induced Animal Model of Achilles Tendinitis. Int. J. Mol. Sci..

[188] Min J. (2016). Nanolayer Multi-Agent Scaled Delivery from Implant Surface. Ph.D. Thesis.

[189] Fedgchin M., Trivedi M., Daly E.J., Melkote R., Lane R., Lim P., Vitagliano D., Blier P., Fava M., Liebowitz M. (2019). Efficacy and Safety of Fixed-Dose Esketamine Nasal Spray Combined With a New Oral Antidepressant in Treatment-Resistant Depression: Results of a Randomized, Double-Blind, Active-Controlled Study (TRANSFORM-1). Int. J. Neuropsychopharmacol..

[190] Murrough J.W., Charney D.S. (2012). Is there anything really novel on the antidepressant horizon?. Curr. Psychiatry Rep..

[191] Gao Y., Yang C., Wang L., Xiang Y., Zhang W., Li Y., Zhuang X. (2020). Comparable Intestinal and Hepatic First-Pass Effect of YL-IPA08 on the Bioavailability and Effective Brain Exposure, a Rapid Anti-PTSD and Anti-Depression Compound. Front. Pharmacol..

[192] Ueno M., Nakagawa T., Wu B., Onodera M., Huang C.L., Kusaka T., Araki N., Sakamoto H. (2010). Transporters in the brain endothelial barrier. Curr. Med. Chem..

[193] Mintz K.J., Mercado G., Zhou Y., Ji Y., Hettiarachchi S.D., Liyanage P.Y., Pandey R.R., Chusuei C.C., Dallman J., Leblanc R.M. (2019). Tryptophan carbon dots and their ability to cross the blood-brain barrier. Colloids Surf. B.

[194] Che X., Wang M., Wang T., Fan H., Yang M., Wang W., Xu H. (2016). Evaluation of the Antidepressant Activity, Hepatotoxicity and Blood Brain Barrier Permeability of Methyl Genipin. Molecules.

[195] Micó J.A., Ardid D., Berrocoso E., Eschalier A. (2006). Antidepressants and pain. Trends Pharmacol. Sci..

[196] Savalia N.K., Shao L.X., Kwan A.C. (2021). A Dendrite-Focused Framework for Understanding the Actions of Ketamine and Psychedelics. Trends Neurosci..

[197] Aleksandrova L.R., Phillips A.G. (2021). Neuroplasticity as a convergent mechanism of ketamine and classical psychedelics. Trends Pharmacol. Sci..

[198] Patel R.B., Rao H.R., Thakkar D.V., Patel M.R. (2022). Comprehending the potential of metallic, lipid, and polymer-based nanocarriers for treatment and management of depression. Neurochem. Int..

